# Environmental pathways affecting gene expression (E.PAGE) as an R package to predict gene–environment associations

**DOI:** 10.1038/s41598-022-21988-6

**Published:** 2022-11-04

**Authors:** Sachin Muralidharan, Sarah Ali, Lilin Yang, Joshua Badshah, Syeda Farah Zahir, Rubbiya A. Ali, Janin Chandra, Ian H. Frazer, Ranjeny Thomas, Ahmed M. Mehdi

**Affiliations:** 1 The University of Queensland Diamantina Institute, Translational Research Institute, The University of Queensland, 37 Kent St, Woolloongabba, QLD 4102 Australia; 2 Centre for Microscopy and Microanalysis, University of Queensland, St. Lucia, QLD 4072 Australia; 3 QCIF Facility for Advanced Bioinformatics, Queensland Cyber Infrastructure Foundation Ltd, Brisbane, QLD Australia

**Keywords:** Computational biology and bioinformatics, Immunology, Environmental biotechnology, Genomics

## Abstract

The purpose of this study is to manually and semi-automatically curate a database and develop an R package that will act as a comprehensive resource to understand how biological processes are dysregulated due to interactions with environmental factors. The initial database search run on the Gene Expression Omnibus and the Molecular Signature Database retrieved a total of 90,018 articles. After title and abstract screening against pre-set criteria, a total of 237 datasets were selected and 522 gene modules were manually annotated. We then curated a database containing four environmental factors, *cigarette smoking, diet, infections* and *toxic chemicals*, along with a total of 25,789 genes that had an association with one or more of gene modules. The database and statistical analysis package was then tested with the differentially expressed genes obtained from the published literature related to type 1 diabetes, rheumatoid arthritis, small cell lung cancer, COVID-19, cobalt exposure and smoking. On testing, we uncovered statistically enriched biological processes, which revealed pathways associated with environmental factors and the genes. The curated database and enrichment tool are available as R packages at https://github.com/AhmedMehdiLab/E.PATH and https://github.com/AhmedMehdiLab/E.PAGE respectively.

## Introduction

Organisms are constantly being exposed to a wide range of environmental triggers that influence gene expression, resulting in several diseases. Environmental factors, such as drugs, toxic chemicals, smoke, temperature, dietary components and infections are considered modifiable causes of disease through their effects on biological processes, and in response, the expression of many genes is altered^1^. It is estimated that environmental factors account for approximately 70% percent of all autoimmune diseases and 80% of all chronic diseases^2^. These large proportions indicate that environmental exposures are an important contributor to disease, and there is ample evidence to support complex interrelationships between various environmental and genomic factors for disease causation^3^. Manipulation of environmental triggers and the host immune system during the clinical and preclinical stages of a disease will offer significant insight and guide early intervention for many disorders^4^.

In the era of Big Data technologies, several genomic databases exist to explore differential expression of genes under various clinical conditions^5,6^. However, to our knowledge there is currently no computational tool that can use information from existing large-scale databases to predict gene–environment relations. Therefore, in this study we formulated an integrated and comprehensive database that will provide insights of how environmental factors are associated to gene expression and disease, and leading to the identification of potential therapeutic strategies for the prevention and control of diseases attributable to both environmental and genetic factors.

## Methods

We followed a two-step approach to conduct this study. First, we conducted a systematic review using a standard approach to identify all studies that used integrated datasets containing comprehensive information about environmental and genetic risk factors for various diseases. Second, we curated a database and developed a statistical analysis package to enable the user to understand the relationships between differentially expressed genes and select environmental factors.

### Step 1: Systematic review

The aim of this step was to identify the relevant published literature from where we could obtain existing data pertinent to gene expression changes in response to an environmental factor. In detail the systematic review was conducted as follows:

### Search strategy

We undertook a comprehensive literature and database search using PubMed, Gene expression omnibus (GEO), and Gene set enrichment analysis (GSEA) databases^7^. All databases were searched from their inception until 16th October 2020. The reference lists of all the retrieved studies were examined to identify additional studies.

The search terms and their synonyms related to environmental factors and gene expression. The keywords used included medical subject headings (MeSH) terms, e.g., ("Diet"[MeSH Terms] OR diet [All Fields]) AND ("gene expression"[MeSH Terms] OR gene expression [All Fields]). Table 1 details the search strategy and date of searches for various databases.

**Table 1:** Search strategies used for database searching.

Search term	Number of hits (total)	Date of search hits
Cigarette smoking AND Gene expression	324	16/10/2020
Diet AND Gene expression	25,440	16/10/2020
Infection AND Gene expression [GEO Database]	59,338	16/10/2020
C7 Immunologic gene sets [GSEA]	4872	16/10/2020
Toxic chemical AND Gene expression	44	16/10/2020

#### Inclusion/exclusion criteria

Pre-set inclusion criterion for studies to be considered eligible were:

- Only articles written in English
- Participants of any age group and both genders.
- Since most of the experimental trials involving environmental factors were carried out in humans or mice, we included hits for Homo sapiens and Mus musculus.
- Four specific environmental factors were chosen, based on the previous published evidence for major contribution as an environmental factor affecting gene expression^8^. Specifically,
  o Cigarette smoking—Includes data related to the practice of tobacco smoking and inhalation of tobacco smoke.
  p Diet—Includes data on the various types and quantities of food consumed by a person.
  q Infections—Includes data on infections caused by pathogenic organisms such as viruses, bacteria, fungi, protozoa and parasites.
  r Toxic chemicals—Includes data on substances such as metals or other chemical agents that are hazardous to human health if inhaled, ingested or absorbed.
- We included published data from datasets, series and platforms. Samples were excluded if they consisted of unpublished data. We did not limit the search specific for any disease.

We did not include any dataset relating to mRNA, protein, CDS or small non-coding RNAs like miRNA or siRNA.

### Literature review method

Two reviewers SM and SA screened the abstracts and citations independently at the same date and time and using the same search parameters. We identified articles that met the inclusion criteria. After title and abstract screening, studies were selected for full-text review. After the full length article review, those studies that met the inclusion criteria were selected for data extraction^7^.

#### Harmonization step

Names of Differentially expressed genes were extracted from GEO and MSigDB C7 databases. Pre-set inclusion criteria were used to select studies to be included in the database. Overlapping studies from the two databases were considered and coded as one study into the spreadsheet. We have further illustrated the harmonization steps in Supplementary SP4 and Figure S1.

### Data extraction

Two reviewers SM and SA independently extracted data. The specific features extracted from each article were: (1) Differential gene expression data; (2) specific description of the type of data collected; (3) specific keywords related to the differentially expressed genes for each dataset, including disease, sample condition and pathways. These were manually searched in the abstract, demographics and result sections of each publication.

#### Data coding

Data were extracted and coded in a spreadsheet to collate information from each study. The data were combined and any anomalies between reviewers were resolved by a third reviewer (LY).

Differential gene expression data were obtained from the results section as well as from the supplementary section of the article. The differentially expressed genesets were annotated based on the information provided in the results section of the article on specific biological processes and/or molecular function regulated. The differentially expressed genes were coded into a spreadsheet and each geneset was provided a unique geneset number. In another spreadsheet the same geneset numbers were provided with annotations extracted from the article and a short description was given to describe the geneset module.

To remove potential bias of manual annotations, E.PAGE also provides functionality to annoSP1tate each geneset using GO, KEGG and MeSH annotations^9–11^ and users have an option to use either of methods or all. Further description on manual curation is provided in supplementary .

### Quality and data validity assessment

The methodological quality was checked before including the data, using the Q-Genie tool^12^. We recorded whether the study used a standard microarray procedure and descriptions of the sample data, causes of up- and downregulation of genes and any other specific changes in the gene expression.

### Step 2: Software generation

The statistical analysis package E.PAGE (Environmental Pathways Affecting Gene Expression) (https://github.com/AhmedMehdiLab/E.PAGE) was written in R version 4.0.3^13^ and developed using RStudio^14^. Using publicly available packages tidyverse^15^ , Seurat^16^ as dependencies, the package performs enrichment analysis as previously described by Mehdi and colleagues^17^.

Mathematically, we represent the collection of annotated modules as ${\mathrm{M}=\{\mathrm{m}}_{1},{\mathrm{m}}_{2},\ldots{\mathrm{m}}_{\mathrm{n}}\}$ and the universal set of genes (background) as $\mathrm{U}=\{{\mathrm{g}}_{1 } ,{\mathrm{g}}_{2 },..{\mathrm{g}}_{\mathrm{w}}\}\mathrm{ with}\, \mathrm{total}\, \mathrm{of}\, \mathrm{w}(\mathrm{U})$ genes. For each query list of genes $\mathrm{g}\subseteq \mathrm{U containing n}(\mathrm{g})\mathrm{ genes}\,\mathrm{ in}\, \mathrm{query}\, \mathrm{list},$ we perform statistical enrichment of each module $\mathrm{m}$ where $\mathrm{m }\in \{{\mathrm{m}}_{1},{\mathrm{m}}_{2},..{\mathrm{m}}_{\mathrm{N}}\}$ with ${\mathrm{N}}_{\mathrm{m}}^{\mathrm{tot}}$ genes associated with $\mathrm{m}$. We compared the number of genes ${\mathrm{N}}_{\mathrm{m}}^{\mathrm{g}}$ that had a specific annotation for gene module ${\text{m}}$ against those that did not. A hypergeometric distribution was used to determine a probability (p-value) that ${\mathrm{N}}_{\mathrm{m}}^{\mathrm{g}}$ or more belong to the module ${\text{m}}$ can be calculated using fisher exact test^18^. The p-value was corrected using false discovery rate (FDR) for multiple hypothesis testing using the Benjamini and Hochberg correction method^19^ to determine the adjusted p-value (${p}_{adj}$). The results are filtered based on the ${p}_{adj}$ are displayed to the user. Fold enrichment was calculated by taking the ratio of a set of genes containing a specific gene modules, and the total set of genes was obtained by taking the union of all the collected gene modules^17^ as follows; $F.E = \frac{{N_{{gm}} /\user2{n}\left( \user2{g} \right)}}{{\user2{N}_{\user2{m}}^{{\user2{tot}}} /\user2{w}\left( \user2{U} \right)}}$. The adjusted fold enrichment was measured as a ratio of the fold enrichment value to the negative log of ${p}_{adj}$. An odds ratio then was measured to determine the probability of finding the set of enriched genes specific to an gene module^20^. We determined the percentage of interactions for four environmental variables (${I}_{\widetilde{m}})$ where $\widetilde{m}=\{\mathrm{cigarette}\,\mathrm{smoking }, \mathrm{diet}, \mathrm{infections },\mathrm{toxic}\, \mathrm{chemicals}\}$, $\widetilde{m}\subseteq {\varvec{M}}$**,** as follows; ${\mathrm{I}}_{\widetilde{\mathrm{m}}}=\frac{{\mathrm{N}}_{\widetilde{\mathrm{m}}}^{\mathrm{g}}}{{\mathrm{N}}_{\widetilde{\mathrm{m}}}^{\mathrm{tot}}}\times 100$. We have provided examples of running E.PAGE in supplementary SP2.

### Step 3: Case studies

We used six case studies to test our enrichment tool, these studies were not used in database curation. Case study 1 involves gene expression data in peripheral blood mononuclear cells (PBMC) in children with type 1 diabetes^21^. Gene expression changes were identified using microarray analysis from 43 patients with new onset T1D compared with 24 healthy controls. The gene expression data set in case study 2 is taken from the GEO database (microarray datasets; GSE12021, GSE55457, GSE55584 and GSE55235) that includes samples from 45 patients with rheumatoid arthritis, compared with 29 healthy control samples^22^. Case study 3 includes gene expression data from 23 small cell lung cancer samples and 42 healthy lung tissues^23^. The gene expression data from the case study 4 was taken from cobalt-exposed rat liver derived cells^24^. The final two case studies used differentially expressed genes extracted from single-cell expression data. Case study 5 was based on single-cell RNAseq data from COVID-19 patients, comparing severe and healthy cases in peripheral immune environments^25^, while case study 6 was based on a single-cell RNAseq-based atlas of epithelial cell-specific responses to smoking^26^. For single-cell RNA seq data, E.PAGE used a Seurat object (with clustering performed) as an input and performs differential expression analyses between the clusters to uncover lists of genes to compute related enriched gene modules.

## Results

### Systematic review and E.PAGE structure

**Figure 1:**
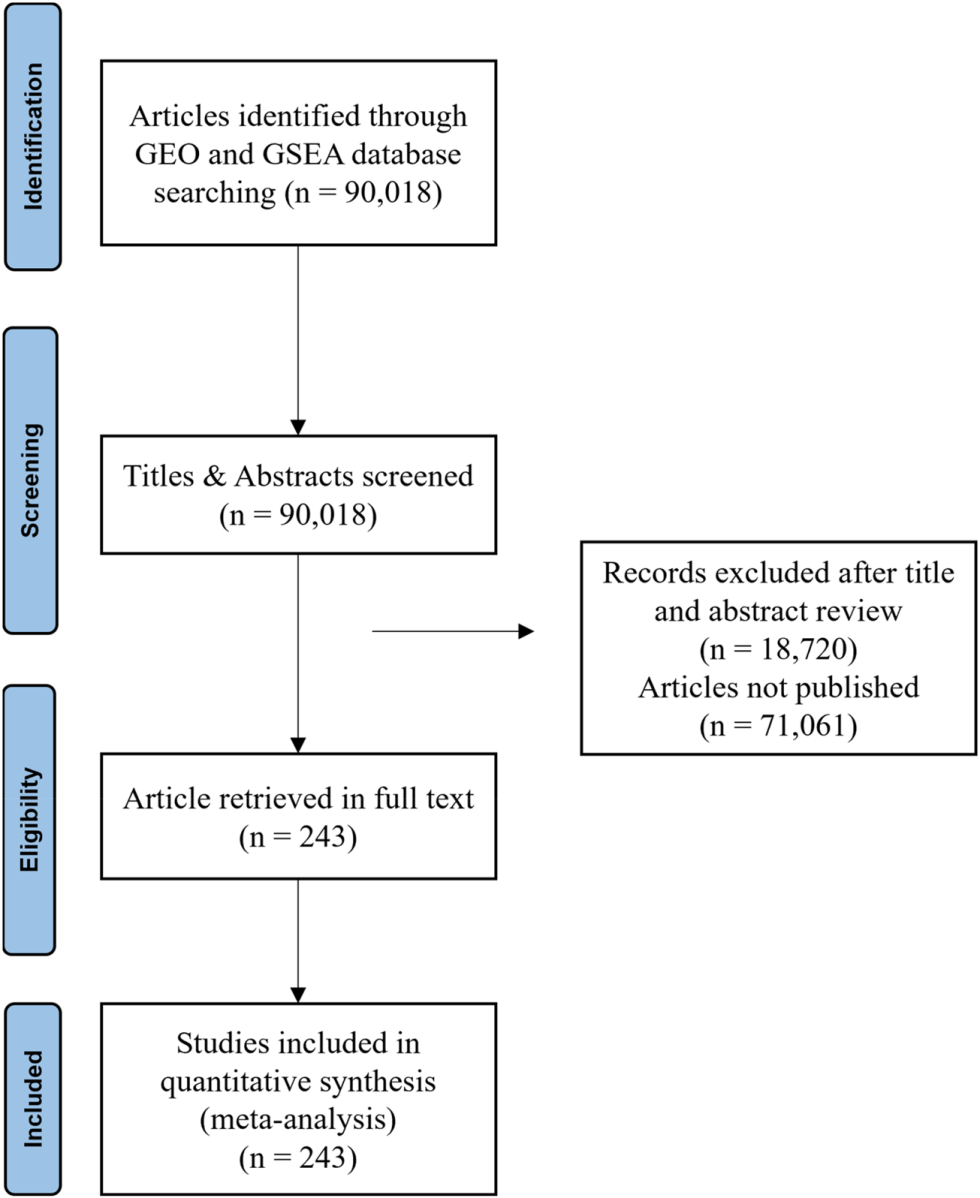
PRISMA flow chart representing the various stages of screening involved in the systematic review process.

**Figure 2:**
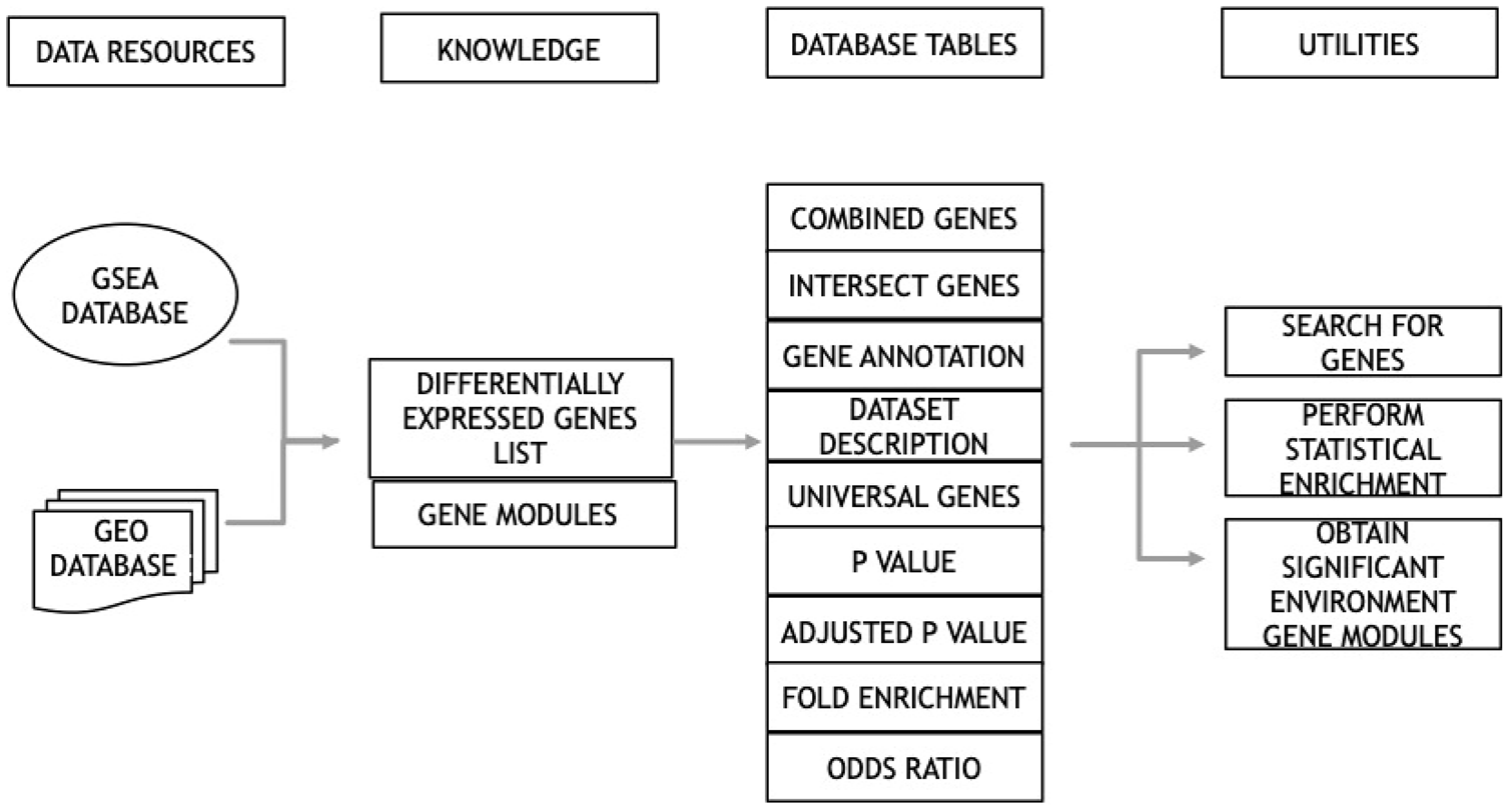
Flow chart representing the various parameters and their utilities provided on database query.

The initial electronic search of GEO and MSigDB database identified a total of 90,018 studies (Fig. 1). Title and abstract screening of retrieved studies resulted in a total of 3547 studies which had potential data related to environmental factors. After full text examination of 3547 studies, 3008 studies were excluded since they did not provide any differential gene expression data associated with any of the four environmental factors. A total of 237 datasets were obtained from 186 studies and the gene expression data were retrieved and collated to form a database. Figure 1 illustrates a flow chart of all the steps taken to obtain the data that satisfy the required parameters. The overall structure of E.PAGE is shown in Fig. 2. After literature screening, a database of 237 datasets was developed by linking each dataset with published lists of differentially expressed genes and the gene modules. Specifically, the text of these 186 publications and associated datasets were manually screened to develop gene modules representing the type of experiment, experimental conditions or disease type, experimental factors, demographics of subjects, and published pathways as previously described by Mehdi and colleagues^17^. The final database consisting of 237 datasets is obtained through GEO and MSigDB databases and includes 18,015 genes for *diet*, 13,259 genes for *infections*, 3841 genes for *cigarette smoking* and 644 genes for *toxic chemicals*.

### Querying E.PAGE

An R package was developed to enable statistical enrichment and gene modules associated with datasets/genes of interest to a user. The package produces various data tables as shown in Fig. 2 and a user can search genes of interest for their statistical enrichment. To test the utility of the statistical analysis package, we performed six case studies as described hereafter.

### Case studies 1 and 2: Immune response activation in type-1 diabetes and rheumatoid arthritis

We first tested whether query signatures associated with T1D and RA could recover common pathways associated with these autoimmune disease. We used 291 DE genes uncovered from 43 patients with new-onset T1D as compared to 24 healthy controls^8^ (Table 2) and 229 DE genes from 45 samples from patients with RA, compared with 29 healthy control samples^22^ (Table 3). The statistical enrichment using E.PAGE identified that the genes in both datasets are involved in *Immune response*. Other significant gene modules that were common to both diseases include *Interferons*, *IL-12* and *Transcription regulation*. These processes are all well known to be involved in RA and T1D^27^. *Insulin resistance* and *Xenobiotic metabolism,* which are both believed to be associated with T1D, were uncovered using E.PAGE and validate the utility of the platform (Table 2). Similarly, for RA, many smoking related gene modules such as Smoking history and Pack years (*Smoking Status: Current, Never*, *Pack-years: (10–20)*, *Pack-years: (20–30; Healthy smoker)*, *(Above 40; Smoker with COPD)*), were uncovered indicating an important risk factor for this disease (Table 3). For both T1D and RA, a large number of gene modules related to infections, both viral and bacterial (*Lyme disease*, *Borrelia burgdorferi*, *HBV Infection*, *Viral response*, *Bacterial infection*, *Zika virus*, *Influenza A Infection*, *HIV infection*, *Echovirus-30*, *Rhinovirus infection*), were significantly associated with disease, indicating that similar responses are occurring in patients suffering from these chronic autoimmune diseases as in responses to infections.

**Table 2:** Collation of results obtained on query of E.PAGE with genes differentially expressed in Type 1 Diabetes.

Gene modules	Number of modules	Number of DE genes	${p}_{adj}$	Fold enrichment
Dyslipemia	1	13	1.46E−08	12.88
Olive oil induced gene expression	3	15	7.52E−08	8.58
Diet intake: Olive oil	2	14	1.14E−07	8.96
Inflammation	31	82	6.87E−07	1.85
Infection type: Acute	58	112	7.46E−07	1.61
Transcription regulation	11	33	8.00E−07	3.09
Interferons	15	53	1.66E−06	2.22
IL-12	4	37	5.02E−06	2.60
Th1-mediated response	4	37	5.02E−06	2.60
Parasite killing	4	37	5.02E−06	2.60
Non-smoker vs Smoker	16	46	3.21E−05	2.13
Type 2 Diabetes	5	16	5.99E−05	4.24
Early Disseminated	1	12	1.11E−04	5.35
Immune response	46	83	1.20E−04	1.59
Cigarette smoking	36	61	1.57E−04	1.77
Monocytes	10	34	2.21E−04	2.26
Airway epithelium	26	58	2.21E−04	1.78
Reactive oxygen species	12	58	2.69E−04	1.76
Mycobacterium tuberculosis	3	14	3.67E−04	3.97
Smoking Status: Current, Never	23	36	6.22E−04	2.08
Chronic obstructive pulmonary disease	16	30	6.36E−04	2.26
Polymorphonuclear leukocytes	10	55	1.08E−03	1.70
Anaplasma phagocytophilum	10	55	1.08E−03	1.70
Granulocytic anaplasmosis	10	55	1.08E−03	1.70
Metabolism	7	35	1.27E−03	2.01
Epithelial gene expression	16	36	1.27E−03	1.98
Lyme disease	2	15	1.27E−03	3.24
Borrelia burgdorferi	2	15	1.27E−03	3.24
PBMCs	22	58	1.27E−03	1.65
DE genes expressed in Obese, Lean	3	94	2.28E−03	1.39
Obese vs Lean	2	94	2.28E−03	1.39
Apoptosis	34	85	4.29E−03	1.40
Protein catabolism	2	10	5.26E−03	3.63
Plasmodium falciparum	1	14	5.26E−03	2.83
Malaria	1	14	5.26E−03	2.83
Blood monocytes	1	14	5.26E−03	2.83
Hepatocellular carcinoma	1	29	5.26E−03	1.95
HBV Infection	1	29	5.26E−03	1.95
Infection type: Chronic	29	66	5.60E−03	1.47
Infection induced gene expression	110	148	6.10E−03	1.21
Pack-years: (10–20)	5	14	6.83E−03	2.74
Diet intake: Dietary energy restriction	3	25	7.21E−03	2.01
DE genes expressed in Obese	15	33	7.27E−03	1.80
Idiopathic pulmonary fibrosis	1	13	7.33E−03	2.82
Cytokines	4	12	1.00E−02	2.83
Lung cancer	8	23	1.00E−02	2.01
Viral response	9	25	1.00E−02	1.94
Mannose metabolism	1	88	1.00E−02	1.34
Insulin resistance	7	89	1.00E−02	1.33
Adipose tissue gene expression	3	88	1.05E−02	1.33
DE genes expressed in Healthy	11	31	1.09E−02	1.77
Before vs After diet intake	7	19	1.23E−02	2.13
Blood immune cells	20	37	1.23E−02	1.65
Influenza A Infection	20	37	1.23E−02	1.65
E. coli infection	20	37	1.23E−02	1.65
Staphylococcus aureus infection	20	37	1.23E−02	1.65
Streptococcus pneumoniae infection	20	37	1.23E−02	1.65
T effector cells	2	11	1.39E−02	2.82
Helminth infection	2	11	1.39E−02	2.82
Macrophages	17	49	1.53E−02	1.50
Lipid metabolism	9	33	1.58E−02	1.68
Infection induced gene expression in mice	18	39	1.65E−02	1.58
Dendritic cells	20	73	1.65E−02	1.35
Vascularization	1	13	1.77E−02	2.42
Energy restriction associated gene expression	2	22	1.77E−02	1.90
Oxidative stress	11	24	1.89E−02	1.82
Hematopoiesis	2	13	1.95E−02	2.38
Vesicular traffic	1	12	2.10E−02	2.43
DE genes expressed in Insulin sensitive individuals	1	12	2.10E−02	2.43
Lipid induced gene expression	1	15	2.22E−02	2.15
Xenobiotic metabolism	4	13	2.66E−02	2.26
Bacterial infection	4	13	2.82E−02	2.24
Protein Metabolism	2	12	3.00E−02	2.31
Skeletal muscle gene expression	2	12	3.03E−02	2.30
Maternal cigarette smoking	2	13	3.03E−02	2.20
Mosquito-borne pathogen	7	21	3.58E−02	1.77
Signal Transduction	7	15	3.62E−02	2.01
Zika virus	8	21	4.12E−02	1.75
Pack-years: (20–30; Healthy smoker), (Above 40; Smoker with COPD)	4	12	4.50E−02	2.15

**Table 3:** Collation of results obtained on query of E.PAGE with genes differentially expressed in Rheumatoid Arthritis.

Gene modules	Number of modules	Number of DE genes	${p}_{adj}$	Fold enrichment
Infection type: Acute	58	131	8.49E−34	2.63
Immune response	46	107	1.03E−27	2.87
Infection induced gene expression	110	159	1.03E−27	1.82
Inflammation	31	91	5.26E−22	2.88
PBMCs	22	78	6.89E−20	3.10
Transcription regulation	11	44	1.16E−19	5.77
Interferons	15	63	3.98E−19	3.70
Central nervous system	4	27	4.96E−18	10.49
Infection type: Chronic	29	85	5.19E−18	2.65
Astrocytes	2	17	1.94E−17	24.50
Plasmodium falciparum	1	29	9.43E−17	8.21
Malaria	1	29	9.43E−17	8.21
Blood monocytes	1	29	9.43E−17	8.21
Dendritic cells	20	91	1.85E−16	2.36
Mycobacterium tuberculosis	3	25	2.53E−16	9.93
Infection induced gene expression in mice	18	59	4.97E−16	3.35
Pro-inflammatory response	1	15	1.72E−15	24.29
Chemokines	2	17	3.11E−15	17.76
Viral response	9	41	9.93E−15	4.46
Monocytes	10	43	7.69E−14	4.00
Olive oil induced gene expression	3	17	2.43E−13	13.61
Bacterial infection	4	27	2.79E−13	6.51
Dyslipemia	1	14	3.35E−13	19.40
Bone marrow monocytes	1	16	3.35E−13	14.78
Myelodysplastic syndromes	1	16	3.35E−13	14.78
Hematopoietic stem cell disease	1	16	3.35E−13	14.78
Lyme disease	2	24	7.14E−13	7.25
Borrelia burgdorferi	2	24	7.14E−13	7.25
IL-12	4	39	4.66E−12	3.84
Th1-mediated response	4	39	4.66E−12	3.84
Parasite killing	4	39	4.66E−12	3.84
Diet intake: Olive oil	2	15	7.67E−12	13.43
Airway epithelium	26	60	2.08E−11	2.57
DE genes expressed in Obese	15	43	3.55E−11	3.28
Blood immune cells	20	48	3.55E−11	2.99
Influenza A Infection	20	48	3.55E−11	2.99
E. coli infection	20	48	3.55E−11	2.99
Staphylococcus aureus infection	20	48	3.55E−11	2.99
Streptococcus pneumoniae infection	20	48	3.55E−11	2.99
Mosquito-borne pathogen	7	34	4.44E−11	4.02
Zika virus	8	34	6.48E−11	3.96
Tissue remodeling	1	10	1.04E−09	19.74
Immunoregulation	1	10	1.04E−09	19.74
Hepatocellular carcinoma	1	36	1.13E−09	3.38
HBV Infection	1	36	1.13E−09	3.38
Chronic obstructive pulmonary disease	16	33	3.61E−09	3.48
Lipid metabolism	9	41	3.98E−09	2.91
Cigarette smoking	36	57	4.61E−09	2.31
Macrophages	17	54	1.51E−08	2.31
HIV infection	9	30	1.59E−08	3.54
Non-smoker vs Smoker	16	42	1.60E−08	2.72
Metabolism	7	37	1.82E−08	2.97
Zika virus associated pDCs response	1	15	3.13E−08	7.21
Early Disseminated	1	13	8.80E−08	8.11
Apoptosis	34	78	9.83E−08	1.80
Type 2 Diabetes	5	16	1.40E−07	5.93
Reactive oxygen species	12	52	1.44E−07	2.21
Fusobacterium nucleatum	3	10	1.58E−07	11.53
Oral pathogen	3	10	1.58E−07	11.53
Diet intake: Low calorie diet	4	23	1.60E−07	3.98
Epithelial gene expression	16	36	1.63E−07	2.77
Smoking Status: Current, Never	23	35	1.67E−07	2.82
DE genes expressed in Healthy	11	35	1.97E−07	2.80
Echovirus-30	1	13	2.33E−07	7.35
Blood‚ÄìCerebrospinal Fluid Barrier	1	13	2.33E−07	7.35
Polar Infection	1	13	2.33E−07	7.35
Skeletal muscle gene expression	2	18	3.23E−07	4.83
Before vs After diet intake	7	23	8.32E−07	3.60
Pack-years: (20–30; Healthy smoker), (Above 40; Smoker with COPD)	4	18	8.72E−07	4.50
T effector cells	2	15	1.08E−06	5.38
Helminth infection	2	15	1.08E−06	5.38
Cell growth	7	15	1.37E−06	5.27
Macrophages gene expression	4	12	1.40E−06	6.89
Cell culture based smoking effect	3	13	1.62E−06	6.12
Cystic Fibrosis	1	24	1.62E−06	3.33
Rhinovirus infection	1	24	1.62E−06	3.33
Human choroid plexus epithelial cells	1	12	1.79E−06	6.70
Cytokines	4	15	2.70E−06	4.96
SIV infection	6	24	2.81E−06	3.22
Weight associated gene expression	10	16	2.90E−06	4.59
Polymorphonuclear leukocytes	10	48	2.90E−06	2.07
Anaplasma phagocytophilum	10	48	2.90E−06	2.07
Granulocytic anaplasmosis	10	48	2.90E−06	2.07
Ulcerative colitis	1	10	3.48E−06	7.92
Crohn's disease	1	10	3.48E−06	7.92
Jurkat cells gene expression	1	10	3.48E−06	7.92
Pack-years: (10–20)	5	16	5.00E−06	4.38
Diet intake: Dietary energy restriction	3	26	5.00E−06	2.92
Viral infection	19	62	6.62E−06	1.78
Signal Transduction	7	19	9.18E−06	3.57
Vesicular traffic	1	15	1.32E−05	4.26
DE genes expressed in Insulin sensitive individuals	1	15	1.32E−05	4.26
Protein Metabolism	2	15	2.30E−05	4.04
Idiopathic pulmonary fibrosis	1	14	2.58E−05	4.26
Lung cancer	8	23	3.20E−05	2.81
Oxidative stress	11	25	3.26E−05	2.66
Non-smoker vs Smoker (Healthy smoker, Smoker with COPD)	11	18	4.67E−05	3.26
Zika virus associated CD4T cell response	1	10	1.23E−04	5.06
Diet intake vs Control	17	19	1.64E−04	2.83
Cytoskeletal function	3	21	1.72E−04	2.64
Pathogen sensing	6	14	3.90E−04	3.23
Antimicrobial defense	6	14	3.90E−04	3.23
Supression of T cell activation	6	14	3.90E−04	3.23
Enhanced bactericidal activity	6	14	3.90E−04	3.23
Inhibition of granuloma destruction	6	14	3.90E−04	3.23
Viral responses	4	11	4.20E−04	3.90
Genotoxic	2	14	4.28E−04	3.19
Carcinogen	2	14	4.28E−04	3.19
Chemical induced gene expression	3	14	5.13E−04	3.13
Energy restriction associated gene expression	2	20	7.40E−04	2.41
Calorie restriction effect on old vs young	1	14	1.07E−03	2.90
Innate Immunity	5	46	1.11E−03	1.63
Regulatory T cells	2	10	1.38E−03	3.60
Immunopathology	2	10	1.38E−03	3.60
Helminth Infection	2	10	1.38E−03	3.60
Insulin resistance	7	67	2.63E−03	1.40
Diet intake: High-fat	13	19	3.42E−03	2.16
Non-genotoxic	1	12	3.47E−03	2.79
Hepatocarcinogens	1	12	3.47E−03	2.79
Liver-based in vitro models	1	12	3.47E−03	2.79
Immune response	2	36	4.56E−03	1.64
Dendritic cell maturation	2	36	4.56E−03	1.64
Newcastle disease virus	2	36	4.56E−03	1.64
Adipose tissue gene expression	3	64	7.85E−03	1.36
Mannose metabolism	1	63	1.14E−02	1.34
Hematogenous dissemination of virus	6	15	1.54E−02	2.04
Epidermal growth factor receptor/PI3K signaling pathway	6	15	1.54E−02	2.04
Obese vs Lean	2	63	1.89E−02	1.31
DE genes expressed in Obese, Lean	3	63	1.90E−02	1.31
Lipid induced gene expression	1	11	2.42E−02	2.21
CD4 + T cell	7	11	2.53E−02	2.19
Pack-years: (20–30)	9	16	3.38E−02	1.80

### Case study 3: Regulation of the cell-cycle process in small cell lung cancer

We next studied gene modules associated with small cell lung cancer. The query signature containing 71 DE genes was derived from 23 clinical small cell lung cancer samples and 42 healthy control tissues^23^. We found that several lungs cancer associated gene modules were infections were was the most common environmental factor associated with the DE genes statistically significant (Table 4). The effect of Cigarette smoking (*Tumor tissue vs Non tumor tissue in Non-smoker vs Smoker, Cigarette smoking, Smoking Status: Current, Never*) was also evident. As expected, *Lung tissue gene expression* and *Adenocarcinoma* were amongst the top five gene modules, along with *Cytoprotective mechanism*, *Mitotic spindle formation genes* and *Cell cycle*, which are important pathways dysregulated in cancer (Table 4). Other interesting gene modules that are known to be involved in lung cancer were also identified, including *Lung cancer, Cigarette smoking, Airway epithelium* and *Immune response*.

**Table 4:** Collation of results obtained on query of E.PAGE with genes differentially expressed in small cell lung cancer.

Gene modules	Number of modules	Number of DE genes	${p}_{adj}$	Fold enrichment
Cytoprotective mechanism	1	21	7.28E−08	5.33
Mitotic spindle formation genes	1	10	1.95E−07	15.80
Cell cycle	4	10	8.49E−07	12.98
Lungs tissue gene expression	2	10	8.49E−07	12.62
Adenocarcinoma	2	10	3.62E−04	6.39
Tumor tissue vs Non tumor tissue in Non-smoker vs Smoker	3	10	6.78E−04	5.82
Apoptosis	34	34	9.36E−04	1.97
Smoking Status: Current, Former, Never	5	10	1.21E−03	5.27
Reactive oxygen species	12	23	1.31E−03	2.47
Polymorphonuclear leukocytes	10	22	2.30E−03	2.39
Anaplasma phagocytophilum	10	22	2.30E−03	2.39
Granulocytic anaplasmosis	10	22	2.30E−03	2.39
Cigarette smoking	36	23	2.30E−03	2.35
Macrophages	17	22	2.38E−03	2.38
Smoking Status: Current, Never	23	14	1.10E−02	2.85
Infection induced gene expression in mice	18	17	1.35E−02	2.44
Lung cancer	8	10	4.05E−02	3.08

### Case study 4: Genotoxicity associated with cobalt exposed gene expression

We next used E.PAGE to understand the gene expression pathways involved in cobalt exposure. We used 27 DE genes uncovered by measuring the effect of cobalt exposure on gene expression in two rat liver derived cell lines using microarray analysis^24^. Cobalt exposed DE genes were associated with chemical induced gene expression. Other significant gene modules include *genotoxicity, carcinogen, non-genotoxic, hepatocarcinogens,* and *liver-based *in vitro* models* (Table 5).

**Table 5:** Collation of results obtained on query of E.PAGE with genes differentially expressed in cobalt exposure.

Gene modules	Number of modules	Number of DE genes	${p}_{adj}$	Fold enrichment
Genotoxic	2	8	1.84E−05	12.09
Non-genotoxic	1	8	1.84E−05	12.33
Carcinogen	2	8	1.84E−05	12.09
Hepatocarcinogens	1	8	1.84E−05	12.33
Liver-based in vitro models	1	8	1.84E−05	12.33
Chemical induced gene expression	3	8	1.84E−05	11.87

### Case study 5: Single-cell COVID-19 dataset

From a single-cell RNA sequencing dataset^25^, we first conducted a standard Seurat pipeline to determine the graph based clusters^16^. We then analysed enrichment of gene modules based on DE genes in Seurat clusters in COVID-19 and healthy cases. As expected, we identified *COVID-19, SARS-COV2* modules. Significant enrichment was also observed for the *Inflammation, Infection-type: Acute, Immune response, Infection induced gene expression* and *Cigarette smoking* amongst the top modules that were previously shown to be COVID-19-related^25,28,29^ (Table 6).

**Table 6:** Collation of results obtained on querying E.PAGE with genes differentially expressed in severe COVID-19.

Gene modules	Number of modules	Number of DE genes	${p}_{adj}$	Fold enrichment
Inflammation	31	188	1.18E−60	3.39
Infection type: Acute	58	225	1.02E−56	2.58
Immune response	46	187	7.61E−49	2.86
Infection induced gene expression	110	273	1.41E−44	1.79
Interferons	15	123	1.89E−43	4.11
Cigarette smoking	36	143	6.85E−41	3.31
Chronic obstructive pulmonary disease	16	90	1.80E−39	5.42
PBMCs	22	139	3.93E−37	3.15
DE genes expressed in Obese	15	99	2.19E−35	4.30
Mycobacterium tuberculosis	3	49	9.60E−35	11.09
Non-smoker vs Smoker	16	105	4.88E−34	3.88
Infection type: Chronic	29	151	3.17E−33	2.69
Monocytes	10	87	6.19E−33	4.61
Macrophages	17	126	5.42E−32	3.08
IL-12	4	83	1.20E−31	4.66
Th1-mediated response	4	83	1.20E−31	4.66
Parasite killing	4	83	1.20E−31	4.66
Viral response	9	78	1.05E−30	4.84
Macrophages gene expression	4	39	4.60E−30	12.77
Lung cancer	8	73	5.74E−30	5.09
Mosquito-borne pathogen	7	74	6.99E−30	4.99
Zika virus	8	74	1.72E−29	4.92
Reactive oxygen species	12	121	1.30E−28	2.93
Diet intake: Dietary energy restriction	3	74	1.55E−28	4.74
Airway epithelium	26	118	4.15E−27	2.88
Plasmodium falciparum	1	46	3.05E−25	7.43
Malaria	1	46	3.05E−25	7.43
Blood monocytes	1	46	3.05E−25	7.43
Metabolism	7	82	4.91E−25	3.76
Pack-years: (10–20)	5	46	1.16E−24	7.19
Polymorphonuclear leukocytes	10	112	7.05E−24	2.76
Anaplasma phagocytophilum	10	112	7.05E−24	2.76
Granulocytic anaplasmosis	10	112	7.05E−24	2.76
Bone marrow monocytes	1	28	1.65E−23	14.75
Myelodysplastic syndromes	1	28	1.65E−23	14.75
Hematopoietic stem cell disease	1	28	1.65E−23	14.75
Apoptosis	34	159	4.18E−23	2.09
Energy restriction associated gene expression	2	64	8.15E−23	4.40
Idiopathic pulmonary fibrosis	1	42	8.63E−23	7.28
Smoking Status: Current, Never	23	78	1.41E−22	3.59
Epithelial gene expression	16	79	5.50E−22	3.47
Dendritic cells	20	144	5.52E−21	2.13
Lyme disease	2	40	7.47E−21	6.89
Borrelia burgdorferi	2	40	7.47E−21	6.89
Hepatocellular carcinoma	1	69	1.70E−20	3.69
HBV Infection	1	69	1.70E−20	3.69
Blood immune cells	20	85	5.45E−20	3.02
Influenza A Infection	20	85	5.45E−20	3.02
E. coli infection	20	85	5.45E−20	3.02
Staphylococcus aureus infection	20	85	5.45E−20	3.02
Streptococcus pneumoniae infection	20	85	5.45E−20	3.02
Chemokines	2	24	5.79E−20	14.29
Central nervous system	4	35	5.94E−20	7.75
Zika virus associated pDCs response	1	32	7.36E−20	8.77
Pack-years: (20–30; Healthy smoker), (Above 40; Smoker with COPD)	4	42	1.05E−19	5.99
Tissue remodeling	1	19	1.94E−19	21.39
Immunoregulation	1	19	1.94E−19	21.39
Sepsis	1	17	7.12E−19	25.40
CD14 + Monocytes	1	17	7.12E−19	25.40
Innate immune response	1	17	7.12E−19	25.40
Fatty acid metabolism	3	17	1.15E−16	19.41
Non-smoker vs Smoker (Healthy smoker, Smoker with COPD)	11	44	4.18E−16	4.54
Bacterial infection	4	38	7.17E−16	5.22
Early Disseminated	1	25	9.19E−16	8.89
Bronchoalveolar epithelium	1	13	1.04E−14	26.06
Olive oil induced gene expression	3	21	5.82E−14	9.59
HIV infection	9	51	8.54E−14	3.44
SARS-COV2	3	18	5.31E−13	10.88
COVID-19	3	18	5.31E−13	10.88
Infection induced gene expression in mice	18	76	5.83E−13	2.46
Astrocytes	2	16	6.21E−13	13.15
Citric acid cycle	1	13	8.34E−13	19.08
Complement system	1	13	8.34E−13	19.08
Diet intake: Milk fat and protein	1	13	8.34E−13	19.08
Apopotosis	1	13	3.39E−12	17.23
Human gingival fibroblasts	2	13	4.17E−12	16.96
Transcription regulation	11	45	6.79E−12	3.37
Diet intake: Olive oil	2	18	9.01E−12	9.19
Oxidative stress	11	50	1.57E−11	3.03
Dyslipemia	1	15	1.60E−11	11.85
Fusobacterium nucleatum	3	16	1.89E−11	10.52
Oral pathogen	3	16	1.89E−11	10.52
Pro-inflammatory response	1	14	2.47E−11	12.93
Atheroscleorsis	1	10	2.67E−11	24.91
Atherosclerotic cardiovascular disease (ASCVD)	1	10	2.67E−11	24.91
Aging	1	10	2.67E−11	24.91
T effector cells	2	26	3.15E−11	5.32
Helminth infection	2	26	3.15E−11	5.32
Smoking Status: Current, Former, Never	5	34	3.20E−11	4.05
Oxidative phosphorylation	3	13	2.25E−10	12.42
Tumor tissue vs Non tumor tissue in Non-smoker vs Smoker	3	31	2.34E−10	4.08
Xenobiotic metabolism	4	30	3.13E−10	4.16
Human choroid plexus epithelial cells	1	20	3.73E−10	6.37
Adenocarcinoma	2	29	5.47E−10	4.19
Pack-years: (20–30)	9	45	8.40E−10	2.89
Regulatory T cells	2	24	8.95E−10	4.93
Immunopathology	2	24	8.95E−10	4.93
Helminth Infection	2	24	8.95E−10	4.93
Cell culture based smoking effect	3	21	1.12E−09	5.64
Hematopoiesis	2	28	1.92E−09	4.09
Signal Transduction	7	33	2.01E−09	3.54
Cystic Fibrosis	1	39	2.33E−09	3.09
Rhinovirus infection	1	39	2.33E−09	3.09
Angiogenesis	2	14	2.35E−09	9.13
Extracellular matrix metabolism	1	10	3.66E−09	15.51
Autosomal-dominant hyper-IgE syndrome	1	10	3.66E−09	15.51
Immunodeficiency	1	10	3.66E−09	15.51
Lipid metabolism	9	58	3.95E−09	2.35
Vascularization	1	27	5.71E−09	4.01
Oxidant-related	2	13	9.38E−09	9.13
Zika virus associated mDCs response	1	19	2.74E−08	5.21
Maternal cigarette smoking	2	27	3.99E−08	3.65
Cell death	1	20	8.38E−08	4.60
Leptin resistance	1	11	9.12E−08	9.62
Weight loss	2	11	3.15E−07	8.53
Gene expression induced due to fasting	3	13	3.26E−07	6.76
Diet intake: Fasting	3	13	3.26E−07	6.76
DE genes expressed in Healthy	11	49	3.99E−07	2.24
Cytokines	4	21	4.55E−07	3.96
Diet intake: Low calorie diet	4	30	5.45E−07	2.96
SIV infection	6	35	5.45E−07	2.68
Zika virus associated CD8T cell response	1	16	1.24E−06	4.78
Type 2 Diabetes	5	19	1.44E−06	4.01
Ulcerative colitis	1	13	1.57E−06	5.87
Crohn's disease	1	13	1.57E−06	5.87
Jurkat cells gene expression	1	13	1.57E−06	5.87
DNA damage	3	10	3.48E−06	7.54
Weight associated gene expression	10	21	4.12E−06	3.44
Obese vs Lean	2	123	4.99E−06	1.46
DE genes expressed in Obese, Lean	3	123	5.04E−06	1.46
Adipose tissue gene expression	3	121	5.21E−06	1.46
Chemical induced gene expression	3	24	5.25E−06	3.06
Insulin resistance	7	122	5.28E−06	1.46
Genotoxic	2	23	1.27E−05	2.99
Carcinogen	2	23	1.27E−05	2.99
Mannose metabolism	1	119	1.31E−05	1.44
Smoking History: > 19 years	2	12	1.59E−05	5.14
Pack-days: (1–1.21)	2	12	1.59E−05	5.14
Calorie restriction effect on old vs young	1	24	1.87E−05	2.83
Diet intake vs Control	17	29	2.83E−05	2.46
Non-genotoxic	1	22	2.84E−05	2.92
Hepatocarcinogens	1	22	2.84E−05	2.92
Liver-based in vitro models	1	22	2.84E−05	2.92
Cell cycle	4	14	3.19E−05	4.11
Zika virus associated CD4T cell response	1	14	3.81E−05	4.04
Viral responses	4	17	3.81E−05	3.43
Cigarette smoking in women	3	13	4.07E−05	4.29
Lungs tissue gene expression	2	14	4.20E−05	4.00
HIV-1 infection	9	30	4.20E−05	2.36
Smoking Status: Current, Former	2	14	5.33E−05	3.90
Tumor tissue vs Non tumor tissue in Current smoker vs Former Smoker	2	14	5.33E−05	3.90
Zika virus induced B cell response	1	14	6.10E−05	3.84
Zika virus associated B cell response	1	14	6.10E−05	3.84
Zika virus associated monocytes response	1	14	6.10E−05	3.84
Mitotic spindle formation genes	1	12	7.90E−05	4.29
Skeletal muscle gene expression	2	19	1.04E−04	2.91
Metabolic pathways	2	10	1.64E−04	4.70
Innate Immunity	5	75	2.77E−04	1.52
Pulmonary nontuberculous mycobacterial disease	1	10	6.57E−04	3.93
T cell signaling	1	10	6.57E−04	3.93
Before vs After diet intake	7	24	1.03E−03	2.14
Protein Metabolism	2	16	2.17E−03	2.46
Vesicular traffic	1	15	3.42E−03	2.43
DE genes expressed in Insulin sensitive individuals	1	15	3.42E−03	2.43
DNA Methylation	5	11	4.73E−03	2.80
CD4 + T cell	7	17	1.54E−02	1.93
Hematogenous dissemination of virus	6	22	2.11E−02	1.71
Epidermal growth factor receptor/PI3K signaling pathway	6	22	2.11E−02	1.71
Cytoskeletal function	3	23	2.54E−02	1.65
Cytoprotective mechanism	1	27	3.10E−02	1.55
Cell-adhesion	3	16	3.67E−02	1.78
Diet intake: High-fat	13	24	4.03E−02	1.56

### Case study 6: Single-cell smoking dataset

As a sixth case study, we attempted to identify enriched gene modules related to smoking using a single cell RNA sequencing dataset which contained data of smokers vs non-smokers^26^. After processing the data using the Seurat pipeline and analyzing the single-cell expression data, gene set enrichment identified *Epithelial gene expression, Cigarette smoking*, *Airway epithelium*, and *Chronic obstructive pulmonary disease* as the top gene modules with highly significant p-values, confirming that smoking-related pathways were correctly predicted using E.PAGE (Table 7). Furthermore, smoking associated with gene signatures of lung-associated diseases such as *Lung cancer*, *Cystic fibrosis*, as well as with *Carcinogen* and respiratory infections such as *Influenza* and *COVID-19*.

**Table 7:** Collation of results obtained on querying E.PAGE with genes differentially expressed in heavy smoking subjects.

Gene modules	Number of modules	Number of DE genes	${p}_{adj}$	Fold enrichment
Epithelial gene expression	16	198	3.07E−87	5.16
Cigarette smoking	36	261	6.38E−85	3.58
Airway epithelium	26	254	7.98E−85	3.68
Non-smoker vs Smoker	16	206	3.08E−81	4.52
Idiopathic pulmonary fibrosis	1	103	2.05E−73	10.60
Chronic obstructive pulmonary disease	16	158	2.05E−73	5.64
Pack-years: (10–20)	5	105	4.99E−71	9.74
Lung cancer	8	136	4.78E−62	5.63
Smoking Status: Current, Never	23	141	3.87E−44	3.85
Pack-years: (20–30)	9	113	3.95E−39	4.31
Infection type: Acute	58	276	3.54E−31	1.88
Infection induced gene expression	110	390	1.15E−30	1.51
Inflammation	31	207	2.15E−30	2.21
Immune response	46	214	1.04E−23	1.94
Infection type: Chronic	29	192	8.61E−23	2.03
Apoptosis	34	233	2.18E−22	1.82
Transcription regulation	11	77	8.42E−20	3.42
Cystic Fibrosis	1	74	1.82E−19	3.48
Rhinovirus infection	1	74	1.82E−19	3.48
Lyme disease	2	48	2.39E−18	4.91
Borrelia burgdorferi	2	48	2.39E−18	4.91
Non-smoker vs Smoker (Healthy smoker, Smoker with COPD)	11	62	4.36E−18	3.79
Lipid metabolism	9	106	4.56E−18	2.55
Reactive oxygen species	12	146	8.89E−18	2.10
PBMCs	22	152	1.48E−17	2.04
Mycobacterium tuberculosis	3	41	1.59E−17	5.51
Pack-years: (20–30; Healthy smoker), (Above 40; Smoker with COPD)	4	51	3.22E−17	4.32
Macrophages	17	143	6.22E−17	2.07
Infection induced gene expression in mice	18	119	6.23E−17	2.29
Interferons	15	115	3.24E−16	2.28
Polymorphonuclear leukocytes	10	138	2.89E−15	2.02
Anaplasma phagocytophilum	10	138	2.89E−15	2.02
Granulocytic anaplasmosis	10	138	2.89E−15	2.02
Central nervous system	4	38	6.33E−15	5.00
Oxidative stress	11	77	7.09E−15	2.77
HIV infection	9	72	9.37E−15	2.88
Signal Transduction	7	55	1.14E−14	3.50
Hepatocellular carcinoma	1	82	1.93E−14	2.61
HBV Infection	1	82	1.93E−14	2.61
Human choroid plexus epithelial cells	1	30	2.52E−13	5.67
IL-12	4	76	1.01E−12	2.53
Th1-mediated response	4	76	1.01E−12	2.53
Parasite killing	4	76	1.01E−12	2.53
Monocytes	10	78	2.14E−12	2.45
Dendritic cells	20	186	3.07E−12	1.63
Smoking Status: Current, Former	2	30	7.44E−12	4.96
Tumor tissue vs Non tumor tissue in Current smoker vs Former Smoker	2	30	7.44E−12	4.96
Bronchoalveolar epithelium	1	13	1.11E−11	15.46
Viral response	9	69	1.16E−11	2.54
Squamous cell lung carcinoma	1	26	2.11E−11	5.51
Smoking Years Quit: > 2 years	1	26	2.11E−11	5.51
Pack-years: (30–40)	1	26	2.11E−11	5.51
Metabolism	7	82	6.06E−11	2.23
Cytoprotective mechanism	1	70	1.35E−10	2.38
Mosquito-borne pathogen	7	63	1.57E−10	2.52
Zika virus associated pDCs response	1	28	2.84E−10	4.55
Zika virus	8	63	2.84E−10	2.48
SARS-COV2	3	19	4.46E−10	6.81
COVID-19	3	19	4.46E−10	6.81
Lungs tissue gene expression	2	27	5.51E−10	4.57
DE genes expressed in Obese	15	82	7.48E−10	2.11
Mucus overproduction	2	18	8.21E−10	7.02
Skeletal muscle gene expression	2	37	1.35E−09	3.36
Cell culture based smoking effect	3	27	2.03E−09	4.30
Obese vs Lean	2	208	2.03E−09	1.46
DE genes expressed in Obese, Lean	3	208	2.06E−09	1.46
SIV infection	6	55	3.91E−09	2.50
Cytokines	4	32	4.68E−09	3.58
Insulin resistance	7	205	4.75E−09	1.45
Adipose tissue gene expression	3	203	5.36E−09	1.46
Mannose metabolism	1	202	6.76E−09	1.45
Smoking Status: Current, Former, Never	5	41	1.02E−08	2.90
Early Disseminated	1	22	2.01E−08	4.64
Blood immune cells	20	88	6.63E−08	1.86
Influenza A Infection	20	88	6.63E−08	1.86
E. coli infection	20	88	6.63E−08	1.86
Staphylococcus aureus infection	20	88	6.63E−08	1.86
Streptococcus pneumoniae infection	20	88	6.63E−08	1.86
Macrophages gene expression	4	22	8.61E−08	4.27
Mitotic spindle formation genes	1	21	8.68E−08	4.45
Genotoxic	2	37	8.68E−08	2.86
Carcinogen	2	37	8.68E−08	2.86
Cell cycle	4	23	1.30E−07	4.01
Chemical induced gene expression	3	37	1.37E−07	2.80
Chemokines	2	16	1.61E−07	5.65
Dyslipemia	1	14	1.75E−07	6.57
DE genes expressed in Lean	3	10	4.35E−07	9.56
Zika virus associated mDCs response	1	23	4.37E−07	3.74
Vesicular traffic	1	31	4.60E−07	2.98
DE genes expressed in Insulin sensitive individuals	1	31	4.60E−07	2.98
Protein Metabolism	2	32	4.60E−07	2.92
Non-genotoxic	1	35	4.60E−07	2.75
Hepatocarcinogens	1	35	4.60E−07	2.75
Liver-based in vitro models	1	35	4.60E−07	2.75
Astrocytes	2	13	7.52E−07	6.34
DE genes expressed in Healthy	11	70	9.81E−07	1.90
Olive oil induced gene expression	3	17	1.08E−06	4.61
Weight associated gene expression	10	30	1.12E−06	2.91
Transport	3	15	1.18E−06	5.19
Diet intake: Olive oil	2	16	1.19E−06	4.85
Diet intake: Low calorie diet	4	41	1.30E−06	2.40
Pro-inflammatory response	1	12	1.39E−06	6.58
Regulatory T cells	2	26	1.39E−06	3.17
Immunopathology	2	26	1.39E−06	3.17
Helminth Infection	2	26	1.39E−06	3.17
Tumor tissue vs Non tumor tissue in Non-smoker vs Smoker	3	34	1.41E−06	2.66
Fusobacterium nucleatum	3	14	1.45E−06	5.46
Oral pathogen	3	14	1.45E−06	5.46
Diffuse large B-cell lymphoma	1	14	1.54E−06	5.42
Germinal center B-cell	1	14	1.54E−06	5.42
DNA repair	1	14	1.54E−06	5.42
Genomic stability	1	14	1.54E−06	5.42
Prostaglandin metabolism	1	10	3.94E−06	7.39
DE genes expressed in Low calorie diet	1	10	3.94E−06	7.39
Epithelial barrier integrity	1	11	3.94E−06	6.54
Cilia beat activity	1	11	3.94E−06	6.54
Cytoskeletal function	3	49	4.67E−06	2.09
Oxidant-related	2	12	2.20E−05	5.00
Diet intake: Dietary energy restriction	3	51	2.20E−05	1.94
Echovirus-30	1	18	2.49E−05	3.44
Blood‚ÄìCerebrospinal Fluid Barrier	1	18	2.49E−05	3.44
Polar Infection	1	18	2.49E−05	3.44
Adenocarcinoma	2	29	3.16E−05	2.49
Human papillomavirus	2	11	4.65E−05	5.06
Zika virus induced B cell response	1	19	6.29E−05	3.09
Zika virus associated B cell response	1	19	6.29E−05	3.09
Ulcerative colitis	1	14	9.73E−05	3.75
Crohn's disease	1	14	9.73E−05	3.75
Jurkat cells gene expression	1	14	9.73E−05	3.75
Viral infection	19	141	1.10E−04	1.37
T effector cells	2	22	1.28E−04	2.67
Helminth infection	2	22	1.28E−04	2.67
Before vs After diet intake	7	38	1.45E−04	2.01
Cell growth	7	22	1.66E−04	2.62
Innate Immunity	5	116	3.50E−04	1.39
Xenobiotic metabolism	4	27	3.92E−04	2.22
Bacterial infection	4	27	4.44E−04	2.20
DNA Methylation	5	18	4.82E−04	2.72
Energy restriction associated gene expression	2	44	4.91E−04	1.80
Pack-years: Above 40	2	10	1.14E−03	3.75
Gene expression induced due to fasting	3	11	1.36E−03	3.40
Diet intake: Fasting	3	11	1.36E−03	3.40
Type 2 Diabetes	5	19	1.47E−03	2.38
Maternal cigarette smoking	2	25	2.60E−03	2.01
Immune reposne	2	89	3.69E−03	1.37
Dendritic cell maturation	2	89	3.69E−03	1.37
Newcastle disease virus	2	89	3.69E−03	1.37
Cell-adhesion	3	28	4.32E−03	1.85
Diet intake vs Control	17	34	4.93E−03	1.71
Viral responses	4	18	5.73E−03	2.16
Hematopoiesis	2	22	8.83E−03	1.91
Zika virus associated CD8T cell response	1	13	1.22E−02	2.31
Calorie restriction effect on old vs young	1	25	1.33E−02	1.75
Vascularization	1	21	1.41E−02	1.85
Host susceptibility	2	16	2.10E−02	1.95
Macrophage activation	2	16	2.10E−02	1.95
Inflammatory diseases	2	16	2.10E−02	1.95
Plasma insulin level	5	12	2.21E−02	2.19
Pathogen sensing	6	22	2.31E−02	1.72
Antimicrobial defense	6	22	2.31E−02	1.72
Supression of T cell activation	6	22	2.31E−02	1.72
Enhanced bactericidal activity	6	22	2.31E−02	1.72
Inhibition of granuloma destruction	6	22	2.31E−02	1.72
HIV-1 infection	9	32	3.48E−02	1.49
Plasmodium falciparum	1	18	3.91E−02	1.72
Malaria	1	18	3.91E−02	1.72
Blood monocytes	1	18	3.91E−02	1.72
Hematogenous dissemination of virus	6	32	4.04E−02	1.47
Epidermal growth factor receptor/PI3K signaling pathway	6	32	4.04E−02	1.47

#### User-defined annotations

The E.PAGE do not currently incorporate genetic data. However, to demonstrate its feasibility, we separately used two genetic datasets^30,31^ associated with Parkinson’s disease (PD) and developed automatic annotations using E.PAGE (Supplementary SP3). An independent transcriptomic dataset associated with PD was queried^32^. We uncovered annotations such as Genetic Association [Parkinson's Disease, GWAS + eQTL] and cellular response to interferon-gamma.

## Discussion

Environmental factors are known to influence the development of disease, with or without combination with genetic factors, however there is currently no curated database and enrichment tool to identify the genes and the corresponding biological processes associated with these environmental conditions. We developed E.PAGE, a database and enrichment tool to understand the gene–environment relationship. Our database was developed based on experimental evidence obtained from the published literature to establish a relationship between environmental factors, differentially expressed genes and specific biological processes associated with the genes.

To set up the database, we used *cigarette smoking, infections, toxic chemicals* and *diet*, as they constitute the primary environmental factors influencing disease outcomes^4^. We made every effort to ensure completeness, accuracy and currency of the database. The current database has 237 datasets which consists of 25,789 genes in total. Traditional methods assume a linear relationship between environment and the genes^33^. In our study, the annotations such as *Cigarette smoking* have a direct relationship with environmental variables whereas the *Viral response* may have direct or indirect relationship with environmental variables depending on each experiment. Thus the annotations included in the study are a combination of linear and non-linear environment variables^33^. The largest number of datasets relate to *diet* and *infections* due to the long research history of these two environmental factors and disease. We manually curated each dataset using specific keywords and a brief description, abstract published with these datasets. We then developed an enrichment tool that uncovers modules associated with genes of interest using the methods we previously published^17^. In six case studies, we tested E.PAGE with sets of DE genes available from the literature. Specifically, we tested two gene lists associated with autoimmunity—T1D and RA—along with those related to small cell lung cancer, COVID-19 and smoking subjects. To confirm the effect of toxic chemicals on differential gene expression, we also used gene expression data from a study on cobalt exposure.

On testing T1D and RA associated DE genes, we found a large number of gene modules related to immune responses, which supports previous studies on how malfunction in the adaptive immune response results in activation of self-reactive T cells. We also obtained a substantial number of environmental modules associated with viral and bacterial infections, which supports recent findings on how bacterial and viral infections are implicated in immune response signaling in autoimmune disease pathogenesis. The T1D and RA associated DE genes were found to be primarily enriched in *infection-*associated gene modules and less in gene modules associated with the environmental factors *diet, cigarette smoking* or *toxic chemicals*. This information supports the hypotheses that infection-associated immune responses are major contributors to the development of T1D and RA^34–36^. A substantial number of genes involved in the central nervous system were also related to RA, consistent with other evidence^37^.

When small cell lung cancer genes were tested, we found a large number of environmental modules for DE genes to be related to *lung cancer*, as expected. We also found an expected link to *cell cycle*, since cell cycle checkpoints are disrupted leading to tumour development and cancer progression. Genes relating to *cytoprotective function*, *mitotic spindle formation* are also generally dysregulated in cancer. Recent studies that show a high incidence of retrovirus in lung small cell cancer suggest a possible direct link between infections and small cell cancer^38^.

To further assess associations between environmental factors with toxic chemicals, we tested genes differentially expressed due to cobalt exposure against the E.PAGE database. On testing, we found the modules *Genotoxicity* and *Carcinogen* to be enriched. We also obtained a substantial number of genes differentially expressed due to toxic chemicals as environmental factors, supporting the validity of the tool to identify potential involvement of toxic chemicals on DE genes involved in critical functions in a relevant datasets.

On testing gene expression data sourced from patients with COVID-19, we found that genes differentially expressed in severe cases were linked to gene modules common between bronchoalveolar and peripheral immune environments^25,29^. This finding shows how the E.PAGE database can be used to find commonalities between two sets of differentially expressed genes, even if they may not have many genes in common.

On testing the single-cell gene expression data for smoking we found gene modules for Cigarette smoking, Airway epithelium, Epithelial gene expression, and Chronic obstructive pulmonary disease. Additional pathways that are well known to be altered by cigarette smoking were identified. Therefore, E.PAGE was able to find relevant significantly enriched gene modules.

From the above case studies, we found that our database is highly reliable and has the potential to establish a link between environmental factors and important biological processes. In the case studies, we generally obtained a higher number of DE genes related to infection as an environmental factor. Though this link with infection may be valid, there is a possibility of dataset bias due to limited type of input data such as gene list, similarities between infection and tissue damage -associated immune responses. Additionally, our study is limited to four types of environmental variables, therefore to increase usage towards wider community more environmental datasets need to be integrated. Our study is limited to the use of MeSH terms to query GEO database for differential gene expression data. Additional statistical tests such as joint odds ratio and interaction odds ratio could be included to increase the statistical representation of the datasets^39^. Our study is currently limited to four types of environmental variables, therefore to increase usage in the wider community more environmental datasets will be integrated over time. Further updates will be the addition of other statistical tests to cover genetic data such as Single Nucleotide Polymorphisms, Copy Number Variants and DNA Methylations^40–42^.

A key benefit of this research is to predict gene–environment interactions to identify novel associations between environmental factors and disease, and to inform hypothesis synthesis and target selection. Thereby, it allows scientists and epidemiologists to dissect which genes may be influenced by environmental exposures in different disease conditions. We illustrate this by using examples from type-1 diabetes, rheumatoid arthritis, small cell lung cancer and COVID-19 datasets.

The current study lends itself to future extension to additional environmental variables such as alcohol, physical activities, life-style factors, along with inclusion of other kinds of genetic data which could facilitate the development of disease risk prediction models. Additionally, variable selection methods could be employed to select candidates for gene–environmental variables associated with the disease^43^.

## Supplementary Information

Supplementary Information.

## Data Availability

The E.PATH is freely available at https://github.com/AhmedMehdiLab/E.PATH.

## Code availability

The R package (E.PAGE) to process E.PATH is available as an R package is openly available at https://github.com/AhmedMehdiLab/E.PAGE.
