# Supplementary material for: Environmental pathways affecting gene expression (E.PAGE) as an R package to predict gene–environment associations
Source: Sci Rep. 2022 Nov 4;12:18710. doi: 10.1038/s41598-022-21988-6 (PMC9636158; doi:10.1038/s41598-022-21988-6)
Supplement: Supplementary file 1 — Supplementary Information. [file 41598_2022_21988_MOESM1_ESM.pdf]

Supplementary information for
Environmental pathways affecting gene expression (E.PAGE) as an R package to predict gene-environment associations

Supplementary data 1 (SP1): Description of Manual Curation

Articles identified from GEO and GSEA databases were screened and included into our study based on the pre-set inclusion criteria mentioned in the methods section. A total of 237 studies were included in our quantitative synthesis process. Differential gene expression data were obtained from the results section as well as from the supplementary section of the article. Manual annotations related to the differentially expressed genesets were obtained based on the information provided in the results section of the article. The differentially expressed genes and associated annotations were coded into two spreadsheets. The first spreadsheet contained the following information –

1) Unique geneset ID number. CSGE_A – Cigarette Smoking Gene Expression, DGE_B – Diet Gene Expression, IGE_C – Infection Gene Expression and TGE_D – Toxic chemical Gene Expression. A,B,C and D refers to the dataset number.
2) Differentially expressed genes.

The second spreadsheet contained the following information –

1) Unique geneset ID number obtained from the first spreadsheet.
2) Annotations
3) Description on individual dataset.
4) Link to the article.

These two spreadsheets were used as the basis for the generation of an R package to query the database and has been made available as data frames in the R Package.

Supplementary data 2 (SP2): Examples of running E.PAGE

Installation

To install this package, run:

```
# install.packages("remotes")
remotes::install_github("AhmedMehdiLab/E.PAGE")
```

Usage

To use the package, run:

```
library(E.PAGE)

# extract gene list from text input
genes <- "ftl ApoE CTSZ"
input <- process_input_text(genes, capitalize = TRUE)

# alternatively, extract gene list from Seurat object (see documentation
for function parameters)
seurat_path <- system.file("extdata", "ex_seurat.rds", package = "E.PAGE")
seurat_obj <- readRDS(seurat_path)
input <- process_input_seurat(seurat_obj, 0)

# compute enriched annotations
results <- compute(input)
```

Statistically enriched annotations are stored in a tidyverse tibble, and can be viewed with:

```
results$stats


# To uncover the % of interaction of each annotation with users' genes use
the `% match` column in results$stats object;

results$stats$`% match`

Roxygen documentation is available for all functions.
```

Environment-specific analysis

```
# To uncover the specific analyses related to each environmental variable
the compute_set function can be used as follows

results <- compute_sets(input)

# Cigarette smoking-specific analyses can be extracted as;

results$CSGE

# Infection-specific analyses can be extracted as;

results$IGE

# Diet-specific analyses can be extracted as;
```

```
results$DGE

# Chemical exposure-specific analyses can be extracted as;

results$TGE
```

Gene Ontology analysis

Analysis can also be performed on Gene Ontology terms if a database is provided:

```
library(org.Hs.eg.db)

genes <- "ftl ApoE CTSZ"
input <- process_input_text(genes, capitalize = TRUE)
results <- compute(input, org_db=org.Hs.eg.db)
```

Custom annotations

This package also supports importing database and annotation files:

```
# example annotation and database file locations
anno_path <- system.file("extdata/ex_anno.csv", package="E.PAGE")
data_path <- system.file("extdata/ex_data.csv", package="E.PAGE")

# import .csv files (see documentation for function parameters)
anno <- import_annotations(anno_path, ",", TRUE, c(2, 4), 5)
data <- import_database(data_path, ",", FALSE, c(2, 4), 0)

# input genes and compute enrichment
genes <- "GENE1 GENE2 GENE3"
input <- process_input_text(genes)
results <- compute(input, anno, data)
```

Auto-generate annotations

GO, KEGG and MeSH ontology terms can be automatically generated from database files:

```
# import and process database file
data_path <- system.file("extdata/ex_data.csv", package="E.PAGE")
data_raw <- import_database(data_path, ",", FALSE, c(2, 4), 0)
data <- process_database(data_raw)

# generate annotations and save to file (see documentation for function
parameters)
# performs enrichment for all gene sets, thus may take a long time with
large databases
auto_anno(data, "GO", limit_universe = FALSE, save = "anno.csv")
auto_anno(data, "KEGG", limit_universe = FALSE, save = "anno.csv")
auto_anno(data, "MeSH", limit_universe = FALSE, save = "anno.csv")
```

Note: Examples of annotation and data files has been provided with E.PAGE under the folder "extdata".

Supplementary data 3 (SP3): Example of creating annotation and database for Parkinson's disease.

We obtained the genetic (genomic-wide association studies, SET_1) and expression quantitative trait loci (eQTL, SET_2) data associated with Parkinson's Disease from the study of Li et al (2) and Pierce S et etl. (3)

Both sets are provided in Table S2.

Below script generates automatic annotations;

Generating Annotations:

To generate the annotations run as follows [please add the correct path]:

```
data_path <- "PATH_To/PD_data.csv"
data <- import_database(data_path, ",", FALSE, c(2, 1567), 0)
synthesize_go_anno(data,limit_universe = FALSE, save="PATH_To /anno.csv")
anno_path <- "PATH_To /anno.csv "
```

Adding geneset information:

We manually added geneset names *Genetic Association [Parkinson's Disease, GWAS]* and *Genetic Association [Parkinson's Disease, GWAS+eQTL]* for SET_1 and , SET_2 respectively to the `anno.csv` file.

Testing:

We then obtained a testing dataset for genes associated with PD risk through the study of Kia et al (4) and used top 35 candidates in the list (including candidates that are repeated).

```
test=read_excel(' PATH_To/ noi200099supp6_prod_1617209045.75516.xlsx')
anno <- import_annotations(anno_path, ",", TRUE, c(2, 13), 5)
data <- import_database(data_path, ",", FALSE, c(2, 1567), 0)
results=compute(process_input_text(test$ID[1:35]),anno,data)
```

Supplementary data 4 (SP4): Data harmonization steps

Names of differentially expressed genes were extracted from GEO and MSigDB C7 databases. Pre-set inclusion criteria were used to select studies to be included in the database. Overlapping studies from the two databases were considered and coded as one study into the spreadsheet. We have illustrated the harmonization steps in Supplementary Figure S1 as provided below;

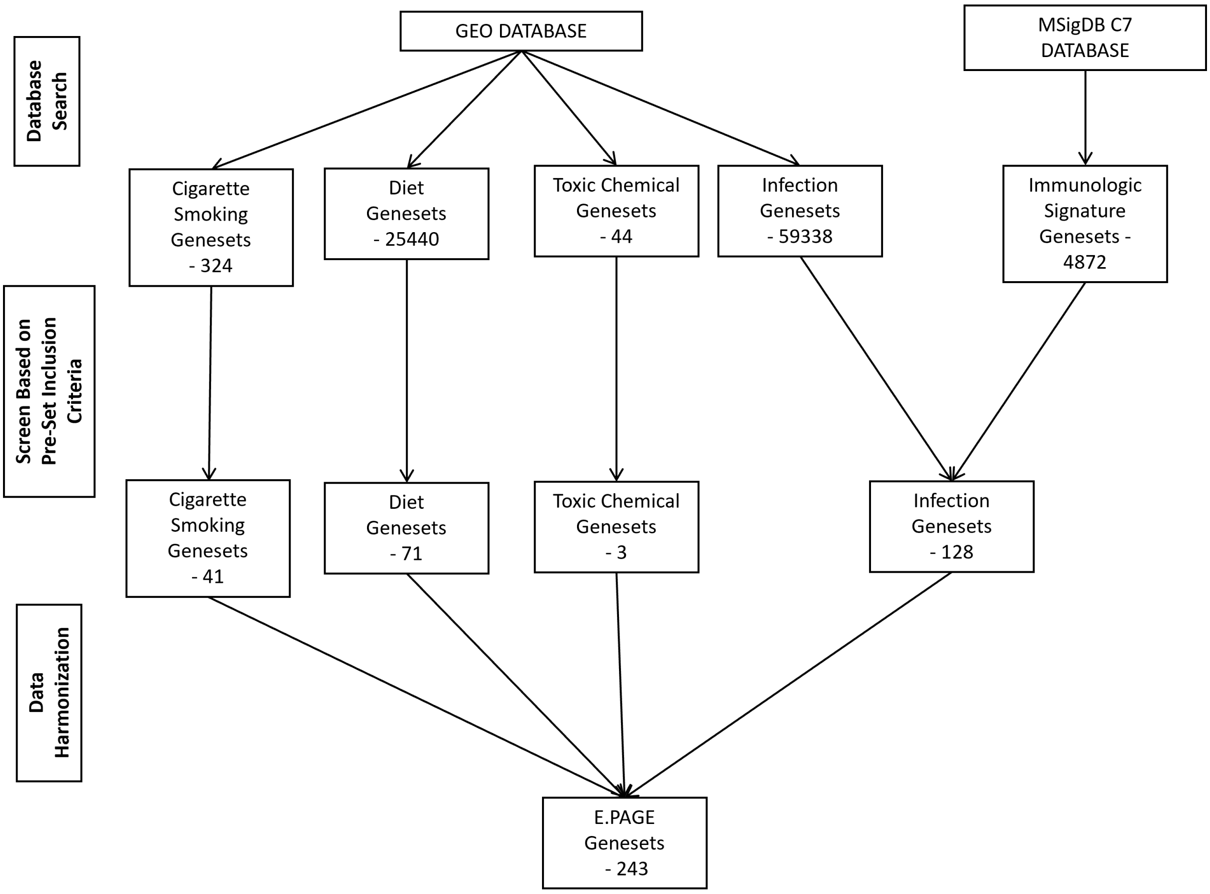

Figure S1: Data harmonization illustrating data retrieved from more than one repositories.

Table S1. Querying E.PAGE on user-defined database for Parkinson's disease

Gene Modules	Number of Modules	Number of DE Genes	Padj	Fold Enrichment
Genetic Association [Parkinson's Disease, GWAS+eQTL]	1	8	0.032	2.2
response to interferon-gamma	1	8	0.032	2.2
antigen processing and presentation of exogenous peptide antigen	1	8	0.032	2.2
antigen processing and presentation of exogenous antigen	1	8	0.032	2.2
antigen processing and presentation of peptide antigen	1	8	0.032	2.2
antigen processing and presentation	1	8	0.032	2.2
cellular response to interferon-gamma	1	8	0.032	2.2
antigen processing and presentation of exogenous peptide antigen via MHC class II	1	8	0.032	2.2
antigen processing and presentation of peptide antigen via MHC class II	1	8	0.032	2.2
antigen processing and presentation of peptide or polysaccharide antigen via MHC class II	1	8	0.032	2.2

Table S2. User-defined database for Parkinson's disease

SET_1	SET_2
GPR126	AC005082.1
SYT11	AC079630.2
SLC2A13	AF131215.2
SLC2A13	ARHGAP27
SLC2A13	ARL17B
SLC2A15	ATP5G2
LINC02471	BIN3
LRRK2	BLK
LRRK2	CCHCR1
GPRIN3	CD38
MAPT	CDK2AP1
LINC02210-CRHR1	CHRNB1
GPRIN3	CLN8
LINC02210-CRHR1	CRHR1
SERPINA1	CRIPAK
SERPINA1	CTD-2303H24.2
S1PR1	CTSB
MAPT	CYP17A1-AS1
MAPT	DGKQ
SPPL2C	DND1P1
LINC02210-CRHR1	FAM106A

LINC02210-CRHR1	FAM167A
HLA-DRA	FAM200B
DGKQ	FAM47E
NSF	FAM47E-STBD1
PRRG4	FDFT1
GAK	FGFRL1
WNT3	GAK
CSMD1	GALC
TAS2R19	GRAMD4
WNT3	HCG27
UNC13B	HLA-B
LINC00693	HLA-DQA1
MPHOSPH10	HLA-DQA2
ZNF519	HLA-DQB1
AAK1	HLA-DQB1-AS1
DHRS2	HLA-DRB1
C12orf75	HLA-DRB5
CDH6	HLA-DRB6
GRB10	KANSL1
PIK3CD	KAT8
SNCA	KLHL7-AS1
LY75-CD302	LACTB
PLA2R1	LINC00926
SH3GL2	LRRC37A4P
PLA2R1	LRRK2
MED13	LSM7
CAST	LTK
LINC01815	LY6G5C
SLCO3A1	MARCKSL1P1
SPPL2C	MCM6
DNAH11	MMRN1
PLA2R1	MPI
LINC01815	MRPS18AP1
PLEKHM1	NAPB
CDH6	NICN1-AS1
LINC01271	NOTCH4
PCAT5	NUCKS1
GABRB3	PPCDC
RNF130	PPM1A
NONE	RAB7L1
LINC01815	RNF40
MCTP2	RP11-115L11.1

LINC01815	RP11-148O21.4
LINC02210-CRHR1	RP11-18I14.10
LINC01815	RP11-244F12.2
DPY19L3	RP11-259G18.3
C20orf78	RP11-282O18.3
HLA-DQB1	RP11-63A1.2
SLC22A3	RP11-665C16.6
SVOPL	RP11-707O23.5
MIR548AG2	RP11-798G7.8
RIPK2	RP11-849F2.8
LINC02210	RP4-756G23.5
MAPT	RPAP1
MAPT	SCARB2
SPPL2C	SHMT1
KCNA1	SLC26A1
ANKRD36	SND1
MED13	SOCS4
MED13	SPTSSB
POMGNT2	STAP1
TMEM2	STK39
43160	STX1B
POMGNT2	TMEM163
SLC2A14	TNFSF12
ABCA3	TOM1L2
SLC2A13	TOP3A
KCNA1	TXNDC15
AP3B1	UBL7
AP3B1	UVSSA
MCL1	VAMP4
LAPTM4A	WNT3
KCNA1	XXbac-BPG248L24.12
TMTC1	XXbac-BPG299F13.17
SND1	C8orf58
LINC00693	CRHR1-IT1
ABCA3	HLA-DQB2
HLX-AS1	NEIL2
LINC02471	NUDT2
BST1	AC005082.12
FER	AC011526.1
SNCA	AC013718.1
GPRIN3	AC016725.4
LOC100133091	AC064850.4

PGGHG	AF131215.9
PRKG1	AL022393.7
GPRIN3	ALG1L13P
CHN2	ARL17A
FER	ATF6B
GAK	ATP1B2
SLC25A48	ATPAF2
SNCA-AS1	B3GALNT1
MAPT	C4B
MAPT	CAB39L
TMEM175	CASC16
MAPT	CCKBR
DGKQ	CTA-398F10.2
KANSL1	CTD-2020K17.1
LINC02210	CTD-2336O2.1
AP3B1	CTD-2514C3.1
CARMIL1	CYP21A1P
CASC6	CYP21A2
CASC6	DDX39B
CASC6	DRG2
CSMD1	ERI1
GPAT3	FAM215B
GPAT3	FAM86B3P
GPAT3	GPNMB
IL2RA	GZF1
IL2RA	HCG23
LINC01572	HLA-C
NDN	HLA-DOB
NDN	HSD3B7
NEDD9	ITPA
NEDD9	KANSL1-AS1
PGGHG	KRT17P2
RBFOX1	LAMP3
RBFOX1	LGALS9C
RGS17	LLGL1
SND1	LRRC37A
SND1	LRRC37A17P
TMPRSS12	LRRC37A2
TMPRSS12	LZTS3
ZNF804B	MAPT
ZNF804B	MCCC1
NUPL2	MCCC1-AS1

SNCA	MYO15A
GPRIN3	NAAA
APOE	NDUFAF1
MAPT	NSFP1
MAPT	NUPL2
TMEM175	PLEKHM1
SNCAdist=796	POU5F1
SNCA	PRRT1
DPM3	PSORS1C2
LRRK2	RNF5
GPRIN3	RP11-101K23.1
SNCA-AS1	RP11-196G11.2
GPRIN3	RP11-259G18.1
MCCC1	RP11-259G18.2
LRRK2	RP11-290F24.3
GBA	RP11-389K14.3
GPRIN3	RP11-62H7.2
STK39	RP11-669E14.6
GPRIN3	RP11-793H13.11
ACMSD	RP11-798G7.5
SNCA	RP11-995C19.2
SNCA	RP5-874C20.3
GPRIN3	RPS26P8
SNCA	SGK223
SNCA	SKIV2L
SNCA	SNORD48
BST1	STX4
FAM47E	TAP2
CCDC62	TCF19
SNCA	TMEM175
RAB7L1	TMEM180
C17orf69	TNFSF13
SNCA	TUBBP5
SNCA	UBXN4
SNCA	VARS2
SNCA	VKORC1
NDUFAF2	XXbac-BPG181B23.7
LRRK2	XXbac-BPG300A18.13
RAB7L1	ZC3H7B
NSF	ZNF204P
CRHR1	ZNF391
GBA	ZNF668

SNCA	
MAPT	
CRHR1	
SPPL2C	
SPPL2C	
KANSL1	
MAPT	
NSF	
CRHR1	
MAPT	
MAPT	
LRRK2	
MAPT	
MAPT	
MAPT	
CRHR1	
CRHR1	
TMEM229B	
MAPT	
CRHR1	
MAPT	
SPPL2C	
KANSL1	
MAPT	
MAPT	
MAPT	
CRHR1	
CRHR1	
CRHR1	
CRHR1	
KANSL1	
LINC02210	
CRHR1	
WNT3	
NSF	
GPNMB	
KANSL1	
CRHR1	
SNCA-AS1	
INPP5F	
GPRIN3	
SH3GL2	

CRHR1	
NSF	
KIAA1267	
MAPT	
SETD1A	
SNCA	
MAPT-AS1	
GPNMB	
HLA-DQB1	
ZNF184	
PARK16	
SLC41A1	
SYT11	
BCKDK	
LRRK2	
DGKQ	
CTSB	
LINC02210-CRHR1	
CCDC62	
RIT2	
SLC2A13	
MIR4697	
RORA	
SLC2A13	
LINC02210-CRHR1	
ITPKB	
LINC02210-CRHR1	
FGF20	
GPRIN3	
FAM47E	
DDRGK1	
GPRIN3	
SNCA	
ANK2	
SNCA-AS1	
SNCA	
SNCA	
MAP4K4	
LINC02210-CRHR1	
GCH1	
SNCA	
NSF	

PLEKHM1	
LINC02210-CRHR1	
LOC201175	
KANSL1	
GPR65	
COQ7	
CASC16	
BST1	
HLA-DRA	
MCCC1	
GPRIN3	
GPRIN3	
SIPA1L2	
DPM3	
FAM47E	
PARK16	
MAPT-AS1	
MCCC1	
WNT3	
BST1	
SNCA	
STK39	
KANSL1	
GPRIN3	
BST1	
SNCA	
GAK	
SLC41A1	
BST1	
NUCKS1	
BST1	
HLA-DRA	
SNCA	
BST1	
SNCA	
IP6K2	
NSF	
HLA-DRA	
LRRK2	
MIR4519	
MCCC1	
NSF	

ITGA8	
GPRIN3	
ITGA8	
LOC101929163	
LOC339862	
SNCA	
BST1	
SLC41A1	
BIN3	
SLC2A13	
MCCC1	
SCN2A	
SNCA	
ITIH1	
GPRIN3	
TMEM175	
SNCA	
LRRK2	
SLC2A13	
SNCA	
PARK16	
DIRC1	
SMCR5	
GPRIN3	
SNCA	
TCEANC2	
SNCA	
FGF20	
NUCKS1	
CYP17A1	
GAK	
KANSL1	
TCEANC2	
TCEANC2	
HLA-DRB5	
SLC2A13	
TCEANC2	
MCCC1	
SLC45A3	
TCEANC2	
PM20D1	
SLC2A13	

SNCA-AS1	
SLC41A1	
LINC02210-CRHR1	
SNCA	
MCCC1	
DLG2	
TMEM59	
NSF	
SLC41A1	
CCDC82	
TMC3-AS1	
LOC100287944	
DGKQ	
RIT2	
RAB38	
BORCS6-ASMT	
MX2	
TCEANC2	
FAM47E	
LDLRAD1	
DCUN1D1	
DLG2	
FAM47E	
LAMP3	
LAMP3	
SNCA	
COL13A1	
SPPL2B	
WBP1L	
PM20D1	
CYCSP42	
LINC02210-CRHR1	
MAPT-IT1	
ZP3	
SMCR5	
CNNM2	
FAM47E	
GPRIN3	
CNNM2	
TCEANC2	
NRIP1	
CCDC62	

PABPN1L	
BORCS7-ASMT	
GFPT2	
PLEKHM1	
SPPL2C	
SPPL2C	
GFPT2	
LINC02210-CRHR1	
GFPT2	
MAPT	
GFPT2	
SNCA	
C17orf69	
BRINP1	
DBC1	
GFPT2	
GFPT2	
MAPT	
NPAS3	
ATP11B	
KCNIP4	
DCUN1D1	
MIR33B	
TCEANC2	
TCEANC2	
TCEANC2	
CNTN1	
SYT11	
KCNIP4	
SNCA	
KCNIP4	
BORCS7-ASMT	
KCNIP4	
C8orf4	
NSF	
NMD3	
SNCA	
MIR663AHG	
SMCR5	
MCCC1	
SREBF1	
TCEANC2	

DIRC1	
LDLRAD1	
TCEANC2	
CNTN1	
RIOK1	
CRHR1	
KCNIP4	
KCNIP4	
KCNIP4	
BST1	
PRF1	
KCNIP4	
KCNIP4	
DGKQ	
MCCC1	
RIOK1	
TCEANC2	
HTR2A-AS1	
LDLRAD1	
SNCA	
KCNIP4	
NUCKS1	
SNCA	
HLA-DRB1	
SNCA	
SNCA-AS1	
MIR663AHG	
KIAA1024	
SNCA	
KCNIP4	
LOC100505817	
HLA-DRB1	
LOC101927620	
GAK	
HFE	
LOC101928985	
MAPT	
C4orf26	
LRRK2	
LINC02064	
SCARB2	
HCG27	

LOC101929163	
DSCAM	
KIAA1274	
SNCA	
STK39	
GAK	
FAM47E	
MCCC1	
CAST	
LOC101929163	
ISM1	
ODF4	
FLJ45872	
LINC00355	
ITGA8	
TOM1L2	
EMR4P	
LOC101929163	
TIAL1	
SNCA	
LRP1B	
SNCA	
KIF4B	
ESRRG	
LOC100129900	
IDO2	
EHMT2	
COL1A2	
HCG27	
SMCR5	
LINC02471	
DCUN1D1	
SEMA5A	
FAM47E	
LRP1B	
FOXK2	
FAM47E	
LMNB1	
LRRK2	
EHMT2	
LRP1B	
CNTN1	

ACTR3P3	
HLA-DQB1	
LOC100131940	
SLC2A13	
DRG2	
RORA	
GPRIN3	
ATPBD4	
TSG1	
C18orf1	
ZFP64	
FLJ23172	
PRDM2	
PDE10A	
PAQR3	
MDGA2	
CLRN3	
SNCA	
HLA-DQB1	
NMD3	
HLA-DQB1	
LINC01363	
SPOCK1	
PRSS16	
TRPM3	
FAM49A	
AGAP1	
CAST	
KREMEN1	
ZBTB20dist=5000	
SNCA	
TTLL5	
LINC02471	
LOC100129831	
ABCA13	
DRG2	
REC114	
PLCB1	
FAM47E	
C17orf39	
KLHL7	
LRRK2	

MCCC1	
PASD1	
LRRK2	
MYO1E	
SLC16A14	
LOC729305	
MAGI2	
TFAP2C	
LINC02210-CRHR1	
RBMS3	
SEMA5A	
TTLL5	
TENM4	
SEPHS1	
TOM1L2	
RPL32P12	
LOC100131940	
SEMA5A	
ARNT2	
LOC646114	
DMRTA1	
PRTFDC1	
MIR100HG	
KLHL7-AS1	
SPEF2	
SNCA-AS1	
LRP1B	
RIT2	
EPB41L3	
HLA-C	
ABI3BP	
FAM126A	
ABCA13	
SUSD3	
GABRB3	
IL5RA	
MIR548AD	
SCARB2	
SNCA-AS1	
LRP1B	
LOC101927450	
VN1R10P	

LINC00707	
LINC00656	
SEMA5A	
WDHD1	
RBFOX1	
MMP16	
SLC16A14	
TOM1L2	
BST1	
LINC02210-CRHR1	
SNCA	
CEP85L	
RAB7L1	
SNCA-AS1	
MYO15A	
TOM1L2	
KDM2B	
MAPT	
KLHL7	
ZNF827	
KLHL7	
ERG	
KLHL7	
FAM171A2	
KLHL7	
LRP1B	
LRP1B	
MUC19	
DCDC2C	
DCUN1D1	
LRP1B	
ZNF596	
SCARB2	
HMGN2P9	
C9orf135	
TMEM163	
FAM171A2	
SLC45A3	
CNTN1	
ACTR3B	
LOC105374704	
KANSL1	

RIT2	
MAPT	
CCNYL2	
KLHL7	
GID4	
SNCA	
SEMA5A	
ARHGAP24	
KLHL7	
WDHD1	
PLCB4	
ITGA6	
SPPL2B	
GAK	
MIR548AD	
MECOM	
SEMA5A	
LARP1B	
LOC729856	
IL5RA	
DSCAM	
SNCA	
CNTNAP5	
ARHGAP24	
CNTNAP5	
CACNA2D1	
SPPL2B	
PTPRT	
C3orf20	
CRHR1	
RPP25	
MVB12B	
TFAP2C	
TMEM200C	
SNX6	
SLC16A14	
CNTNAP5	
LINC00355	
NPTN	
TOM1L2	
TRIM2	
MVB12B	

LINC00355	
ITGA8	
GALNT3	
SYT17	
PSORS1C2	
H1FX-AS1	
SYT17	
SLC5A12	
EIF4A3	
SENP7	
ANK2	
GJB2	
GID4	
TMEM175	
RPS28P6	
MVB12B	
PRDM2	
SLC24A2	
FAM47E	
SCIN	
MIR4493	
ADAMTS6	
MUC19	
HS3ST3A1	
LOC100129138	
CNTLN	
LAMP3	
PHF5GP	
LOC101929066	
HLA-C	
CAVIN1	
HCG27	
NRIP1	
PRKAA1	
TOM1L2	
CNTN1	
LINC02471	
JARID2	
GPRIN3	
GCFC2	
KPNA4	
SYT17	

TRIO	
DCUN1D1	
SNCA-AS1	
TRIML2	
SNCA	
ADAMTS6	
KLHL7	
FTCDNL1	
BTF3P16	
SCAMP5	
KLHL7	
LOC100129900	
SPPL2C	
ACTN4	
SREBF1	
WAC	
PTPRT	
CNTN1	
SNCA-AS1	
PCDH8	
IMPG2	
TIAL1	
GPRIN3	
RETREG3	
SYT17	
FAM167A	
TIAL1	
RORA	
GNAQ	
PTMAP6	
RIT2	
LAMP3	
PASD1	
GAK	
MCCC1	
LRRK2	
SMIM15-AS1	
LRRK2	
FAM18B3P	
HMCES	
KLHL7	
BAG6	

LOC101929163	
ABHD16A	
DAPK1	
LINC00704	
NHLRC3	
COL24A1	
FAM171A2	
LOC100287632	
MAPT	
TMEM163	
FDPSP3	
GAK	
TRIM15	
MOSPD2	
DMRTA1	
DSCAM	
TOM1L2	
MYO15A	
SCARB2	
DRG2	
LOC102724152	
LOC102724152	
BTNL2	
RIT2	
CPNE4	
MBL2	
HLA-C	
KPNA7	
KDM2B	
DFNA5	
FAM47E	
LOC102724152	
SNORA38	
KHDRBS3	
LRRK2	
SLC41A1	
LINC01242	
FRMD4A	
TIMD4	
LOC441666	
LARP1B	
SYT17	

TFAP2C	
MAPT	
PASD1	
LRRK2	
KEL	
MBD5	
LRRK2	
YWHAZP2	
LARP1B	
CRHR1	
RORA	
MBD5	
RIT2	
SLC2A13	
LARP1B	
SLC2A13	
TOM1L2	
TFAP2C	
LRP1B	
SLC16A14	
NTF3	
DRC3	
CRHBP	
KANK1	
FLJ43860	
KIF1BP	
HLA-A	
PARD3-AS1	
BIN3	
COL2A1	
LINC01098	
GRIP1	
LOC645177	
LMO7DN	
CCDC102B	
DOCK5	
LOC100507657	
PLS1	
MCFD2	
PLS1	
CYP1B1	
TACC2	

PAMR1	
DGKQ	
NA	
KANK1	
HDAC7A	
TLE1	
SP140	
TLE1	
GCH1	
TLE1	
KANK1	
STK39	
WNT9A	
ANXA1	
ETV6	
LOC729160	
BIN3	
FAM47E	
FLJ35379	
MMP16	
TBX3	
PLEKHN1	
CCDC149	
SOX5P	
SERPING1	
LOC101927620	
LOC391040	
MRPL50P1	
LINC02405	
DMRT2	
ETV6	
SLC41A1	
CCAR2	
MIR3166	
DKFZp761B107	
ARL6IP6	
SIPA1L2	
LOC101928306	
ZNF396	
C3orf67	
CDH8	
RHEB	

SYN3	
VNN2	
SLC41A1	
STK39	
RNU6ATAC	
SNCA	
JCAD	
TUSC1	
RPH3AL	
LINC02210	
FMN1	
LOC105374060	
DOCK1	
CBARA1	
GPNMB	
NRXN1	
LINC02311	
LINC01679	
PRUNE2	
HPCAL4	
DDAH1	
LRP1B	
RPL10L	
ATR	
CPXM1	
ACMSD	
ACTN1-AS1	
NEUROD4	
RBFOX1	
LINC02224	
MAPT	
LOC105370068	
MAPT-IT1	
NA	
BARX1	
KIF16B	
ANKRD50	
ADGRL2	
MAPT-AS1	
MMRN1	
MAPT	
KANSL1	

MAPT	
MAPT	
SMPDL3A	
CDCP1	
PNPT1	
MAPT-AS1	
MAPT	
MAPT	
KANSL1	
CXXC1	
MAPT	
MAPT	
MCPH1	
LINC01170	
MAPT	
ZMAT3	
CDCP1	
AXIN1	
LRP1B	
SP140	
SNCA	
IL20RA	
PARD3-AS1	
SEMA3E	
MAPT	
CHI3L2	
PARD3-AS1	
LOC284930	
ZNF554	
PROK2	
CXXC1	
MAPT	
ZNF615	
CHSY3	
DDX18	
MAPT	
CXXC1	
LINC02188	
MIR548AD	
CCDC62	
COL5A2	
SPATA21	

LOC100129620	
SLC2A13	
LOC646218	
ANGPT2	
TAP2	
RAD51B	
FBXO34	
MAP2K6	
SNCA	
SNCA	
LOC101927822	
FBXO34	
SASH1	
WNK1	
MAPT	
TIAL1	
DPM3	
GBA	
NOTCH4	
NAGLU	
FCGR2A	
VAMP4	
KCNS3	
KCNIP3	
LINC00693	
KPNA1	
MED12L	
SPTSSB	
LCORL	
CLCN3	
PAM	
C5orf24	
TRIM40	
RIMS1	
FYN	
RPS12	
GS1-124K5.11	
FAM49B	
UBAP2	
GBF1	
RNF141	
SCAF11	

FBRSL1	
CAB39L	
MBNL2	
MIPOL1	
RPS6KL1	
CD19	
NOD2	
CNOT1	
CHRNB1	
UBTF	
FAM171A2	
BRIP1	
DNAH17	
ASXL3	
MEX3C	
CRLS1	
DYRK1A	
SNCA	
TMEM175	
STK39	
TMEM229B	
LRRK2	
BCKDK	
MIR4697	
INPP5F	
RIT2	
GCH1	
SIPA1L2	
TMPRSS9	
MAPT	
ACMSD/TMEM163	
STK39	
SREBF1/RAI1	
MIR4697	
FAM129B	
SNX29	
C5orf52	
STK10	
FAM163A	
NAV2	
LOC392452	
GALNT13	

NFYB	
RBMS3-AS3	
AKR1C4	
GALNTL6	
GALNT14	
DPP6	
CTU1	
CDH8	
DIO3	
SLC25A21	
PPP6R3	
LOC339593	
GALNT14	
C17orf51	
C21orf37	
LRPPRC	
CBFA2T3	
TMEM132C	
SCGB1D4	
TMEM158	
LRPPRC	
ADAM10	
LRPPRC	
EXTL3	
ZNF138	
TTC30B	
HSPH1	
LOC389602	
DUSP26	
SOX17	
RPL32P3	
TMX1	
RABL6	
PPP6R3	
SAYSD1	
SAYSD1	
DIO3	
ANTXR1	
MAP3K2	
FAM129B	
ZNF92	
SNX29	

TBC1D5	
LINC00460	
GRPR	
TBC1D5	
CMAHP	
NUPR1L	
XPO6	
GBE1	
RYR2	
PFKP	
TMEM158	
C21orf37	
PCSK6	
DUSP26	
ZNF138	
CHD9	
HESX1	
EVA1C	
GALNT14	
LOC284080	
CBFA2T3	
CYP39A1	
CYP39A1	
CYP39A1	
CYP39A1	
RNU6-21P	
CA8	
MIR378C	
CDH8	
PLCB1	
FCGR2A	
VAMP4	
KCNS3	
KCNIP3	
LINC00693	
KPNA1	
MED12L	
SPTSSB	
LCORL	
CLCN3	
PAM	
C5orf24	

TRIM40	
RIMS1	
FYN	
RPS12	
GS1-124K5.11	
FAM49B	
UBAP2	
GBF1	
RNF141	
SCAF11	
FBRSL1	
CAB39L	
MBNL2	
MIPOL1	
RPS6KL1	
CD19	
NOD2	
CHRNB1	
UBTF	
FAM171A2	
BRIP1	
DNAH17	
ASXL3	
MEX3C	
CRLS1	
DYRK1A	
SH3GL2	
IL1R2	
SATB1	
FAM171A1	
NCKIPS/CDC71	
ELOVL7	
ITPKB	
BIN3	
SCN3A	
GALC	
COQ7	
ALAS1	
CTSB	
ATP6V0A1	
ANK2/CAMK2D	
DNAH1	

STAB1	
ANK2	
ANK2	
SH3GL2	
NOD2	
SPPL2C/MAPT-AS1	
MCCC1	
TMEM175	
LOC105377329	
LINC00693	
CYP17A1/CYP17A1-AS1	
-	
USP24	
PRKN	
BRINP1	
-	
LOC105377329	
MAPT	
TPKB	
IL1R2	
SCN3A	
SATB1	
NCKIPS	
ALAS1	
ANK2	
ELOVL7	
ZNF184	
CTSB	
BIN3	
SH3GL2	
FAM171A1	
GALC	
COQ7	
TOX3	
ATP6V0A1	
SNCA	
LRRK2	
KANSL1/MAPT	
HLA-DQB1	
LRRK2	
SNCA	
SNCA	

OLFM3	
QSER1	
FSCB	
KCNA5	
SNRK	
KCNA5	
ENSA	
SDC1	
KCNA5	
IPO8	
MIR129-1	
LRRK2	
SNCA	
DTX2P1-UPK3BP1-PMS2P11	
SNCA	
MMRN1	
EPHA7	
EPHA7	
EPHA7	
LOC101928978	
LOC101928978	
LOC101928978	
PWRN4	
PWRN4	
MIR7641-2	
MIR129-1	
MIR129-1	
METTL7A	
SNCA	
STH	
KRTCAP2	
SNCA	
MMRN1	
SNCA	
SNCA	
SNCA	
TMEM163	
SNCA	
FAM47E-STBD1	
HIP1R	
NUCKS1	
ELOVL7	

PARK16	
LINC02210-CRHR1	
LINC02210-CRHR1	
LINC02210-CRHR1	
LINC02210-CRHR1	
LINC02210-CRHR1	
LINC02210-CRHR1	
LINC02210-CRHR1	
MMRN1	
SNCA	
LINC02210-CRHR1	
RAB25	
STX1B	
LINC02349	
SNCA	
FAM47E-STBD1	
SNCA	
CAMK2D	
MMRN1	
ILIR2	
GALC	
TOX3	
SNCA	
SNCA	
KRTCAP2	
FAM47E-STBD1	
LAMP3	
SNCA	
HLA-DRB5	
NCKIPSD	
HLA-DRB5	
FAM171A1	
SNCA	
SATB1	
CD38	
SORBS3	
SCN3A	
ALAS1	
SNCA	
COL3A1	
SREBF1	
SNCA	

MMRN1	
MCCC1	
FAM47E-STBD1	
USP25	
RAI1	
SNCA	
USP25	
HIP1R	
CNOT6	
CNOT6	
DCUN1D1	
SREBF1	
NONE	
RAI1	
TOM1L2	
COL3A1	
LINC02210-CRHR1	
CD38	
ADAMTS14	
LAMP3	
DSP	
RAB7L1	
HLA-DQA1	
NONE	
MTHFS	
FBXO15	
HLA-DQA1	
MIR5580	
LINC00900	
LOC105374704	
FAM47E	
HLA-C	
HLA-DRA	
PRF1	
CERS6	
TASP1	
LINC01052	
FLJ25758	
SGCD	
GPATCH2	
RBMS3	
C8orf4	

HLA-C	
RAI1	
LRRK2	
MCCC1	
44623	
IMPG2	
HLA-DQA2	
CCDC158	
LRRK2	
LINC02349	
SNCA	
LOC100288892	
MANEA	
TSHZ2	
FNDC3B	
KIAA1026	
ARD1B	
HLA-DQA2	
HLA-DQA2	
POM121L2	
GAP43dist=471024	
LRRK2	
LSM7	
DRG2	
SP110	
BMP7	
LOC391636	
SHROOM3	
ABHD17C	
LOC100049717	
LOC646609	
MMRN1	
HLA-B	
IMPG2	
KLHL7	
LOC100288911	
MMRN1	
TLE4	
ZNF204P	
SFMBT2	
NXT1	
SP110	

CD38	
LOC100287632	
SLC41A1	
MMRN1	
LINC01095	
LINC00114	
LOC390800	
FAM87A	
SULT4A1	
ACMSD	
ITGA2B	
NUCKS1	
PDZRN4	
CDH6	
TMPRSS9	
LOC100288911	
PCLO	
LINC02210-CRHR1	
SCAMP5	
BMP7	
L3MBTL4	
CFL2	
SP110	
LINC01052	
LINC01052	
PSORS1C1	
LOC728276	
FIBIN	
CALN1	
FAM47E-STBD1	
ARL4A	
GRAMD1B	
LINC01676	
LASS6	
NAT1	
HLA-B	
ATP6V0A1	
HLA-C	
USP25	
LRRK2	
SNCA	
LRRTM4	

ARL14	
MCCC1	
SENP7	
RBMS3	
RAI1	
BAMBI	
MMRN1	
OLFM4	
LOC100128880	
SNCA	
C8orf12	
LINC02349	
CEP78	
BMP7	
LAMP3	
GABRG3	
AKR1E2	
LHFP	
ODF2L	
MCM9	
GCH1	
TRIM26	
ASB9	
LOC646609	
MEAT6	
MEAT6	
PCDH15	
HLA-B	
MYH16	
MEAT6	
PRRC2A	
LINC01243	
HAVCR1	
BMP7	
OR9A2	
GYPC	
LINC02210-CRHR1	
LINC02349	
BMP7	
SP110	
NCRNA00051	
HCG9	

CUL2	
NONE	
KCTD12	
MN1	
C2orf58	
WNT3A	
LOC100130911	
LOC100132423	
MIR5580	
SMARCA2	
CTSC	
RPRM	
INO80C	
LOC339902	
CDH11	
PRKAG2	
LINC02247	
IGSF11	
NPS	
LINC02301	
MDGA2	
DCAF5	
BRCAT54	
LINC02393	
PTPDC1	
SNRPB2	
FAT4	
LINC01362	
CLVS2	
EFEMP1	
SKA1	
IL22RA2	
CUL2	
CHIAP2	
CUL2	
MIR3201	
SKA1	
HINT1	
LINC02181	
LOC100288911	
HIP1R	
PLPPR4	

MCPH1	
ZFAT	
KRTCAP2	
SYT11	
LOC108783654	
CDC71	
TLR9	
CAMK2D	
SORBS3	
PSMC3IP	
CDC71	
PDLIM2	
TLR9	
ATP6V0A1	
DNAH1	
PDLIM2	
TUBG2	
C8orf58	
DNAH1	
PSMC3IP	
BAP1	
C8orf58	
BAP1	
TUBG2	
PHF7	
PHF7	
NISCH	
NISCH	
STAB1	
STAB1	
ITIH3	
ITIH3	
ITIH4	
ITIH4	

References:

1. Wu C, and Cui Y. A novel method for identifying nonlinear gene-environment interactions in case-control association studies. *Hum Genet.* 2013;132(12):1413-25.
2. Li B, Zhao G, Zhou Q, Xie Y, Wang Z, Fang Z, et al. Gene4PD: A Comprehensive Genetic Database of Parkinson's Disease. *Front Neurosci.* 2021;15:679568.
3. Pierce S, and Coetzee GA. Parkinson's disease-associated genetic variation is linked to quantitative expression of inflammatory genes. *PLoS One.* 2017;12(4):e0175882.

4. Kia DA, Zhang D, Guelfi S, Manzoni C, Hubbard L, Reynolds RH, et al. Identification of Candidate Parkinson Disease Genes by Integrating Genome-Wide Association Study, Expression, and Epigenetic Data Sets. *JAMA Neurol.* 2021;78(4):464-72.
